# IBCar: Potent Orally Bioavailable Methyl *N*-[5-(3′-Iodobenzoyl)-1*H*-Benzimidazol-2-yl]Carbamate for Breast Cancer Therapy

**DOI:** 10.3390/cancers17152526

**Published:** 2025-07-30

**Authors:** Janina Baranowska-Kortylewicz, Ying Yan

**Affiliations:** 1 Department of Pharmaceutical Sciences, College of Pharmacy, University of Nebraska Medical Center, Omaha, NE 68198-6120, USA; 2 Department of Radiation Oncology, College of Medicine, University of Nebraska Medical Center, Omaha, NE 68198-6861, USA; yyan@unmc.edu

**Keywords:** breast cancer, therapy, nontoxic, oral, microtubule-targeted agents, benzimidazole carbamates

## Abstract

**Objectives**: To investigate the efficacy and underlying mechanisms of IBCar’s biological activity in breast cancer models, both in cell culture and in mice, and to compare its effects on cancer versus normal cells. **Methods**: The cytotoxicity of IBCar was evaluated using the MTS assay to assess metabolic activity and the clonogenic assay to determine reproductive integrity. The impact of IBCar on microtubule integrity, mitochondrial function, and multiple signaling pathways was analyzed using Western blotting, microarray analysis, and live cell imaging. The therapeutic effectiveness of orally administered IBCar was assessed in a transgenic mouse model of Luminal B breast cancer and in mice implanted with subcutaneous triple-negative breast cancer xenografts. **Results**: IBCar demonstrated potent cytotoxicity across a diverse panel of breast cancer cell lines, including those with mutant or wild-type *TP53*, and cell lines with short and long doubling times. Comparative analysis revealed distinct responses between normal and cancer cells, including differences in IBCar’s effects on the mitochondrial membrane potential, endoplasmic reticulum stress and activation of cell death pathways. In breast cancer cells, IBCar was cytotoxic at nanomolar concentrations, caused irreversible microtubule depolymerization leading to sustained mitochondrial dysfunction, endoplasmic reticulum stress, and induced apoptosis. In normal cells, protective mechanisms included reversible microtubule depolymerization and activation of pro-survival signaling via the caspase-8 and riptosome pathways. The therapeutic potential of IBCar was confirmed in mouse models of Luminal B and triple negative BC, where it exhibited strong antitumor activity without detectable toxicity. **Conclusions**: These findings collectively support IBCar as a promising, effective, and safe therapeutic candidate for breast cancer treatment.

## Simple Summary

Approximately 30–40% of breast cancer patients treated with conventional chemotherapy experience side effects that significantly diminish their quality of life. The development of effective, nontoxic alternatives remains the leading unmet need in breast cancer care. Here, we describe a novel, safe anti-microtubule agent, methyl *N*-[5-(3′-iodobenzoyl)-1*H*-benzimidazol-2-yl]carbamate (IBCar), which has demonstrated efficacy across a range of in vitro and in vivo breast cancer models.

## 1. Introduction

Breast cancer (BC) is the second leading cause of cancer-related death and accounts for 25% of all cancers in women [1]. Globally, an estimated 2.3 million new cases of BC are diagnosed each year [2,3]. An estimated 316,950 new cases of invasive BC and 59,080 cases of ductal carcinoma in situ are expected to be diagnosed in the United States in 2025. Approximately 42,250 women will die from BC. The likelihood of a woman being diagnosed with BC during her lifetime has increased from 1 in 11 in 1975 to 1 in 8 today [4,5].

The development of metastasis is the most serious aspect of BC sequelae and represents the most difficult stage to treat. In the majority of cases, metastatic breast cancer (mBC) develops after a series of failed therapies, and consequently, metastatic cancer cells predictably acquire resistance to these treatments. Alarmingly, between 2004 and 2021, the incidence of distant-stage (metastatic) disease at diagnosis rose significantly among newly diagnosed breast cancer patients across all age groups, with the most pronounced increase observed in women aged 20–39 years [6].

Metastatic BC is the leading cause of BC-related mortality, accounting for nearly 90% of all deaths from the disease. The median survival of patients with mBC is approximately three years, with little improvement observed over the past two decades [1,7,8]. Despite recent advances, mBC remains incurable. Systemic chemotherapy continues to be the cornerstone of most treatment regimens; however, many drugs eventually lose efficacy due to primary or acquired resistance. This resistance contributes to refractory disease, therapeutic failure, and ultimately, patient death. Overall response rates for second- and third-line therapies in previously treated mBC patients range from approximately 10% to 35% [9,10,11,12].

There is an urgent need for safe and effective therapies for patients with mBC, particularly those whose health and immune function have been compromised by multiple prior treatments. To improve survival outcomes and effectively eliminate breast cancer cells, new therapies must be safer and capable of overcoming drug resistance. Given the clinical success of vinca alkaloids, taxanes, and newer microtubule targeting agents such as eribulin [13,14,15] or ixabepilone [16,17,18], it is evident that microtubules (MTs) remain an excellent target for drug development. Dynamic MTs are assembled from α- and β-tubulin heterodimers, two highly conserved proteins that are present in all eukaryotic organisms. MTs play a fundamental role in diverse cellular functions including cell division, growth and motility [19,20,21,22]. The category of MT-targeted drugs comprises diverse agents that interfere with MTs dynamics, by either stabilizing or destabilizing tubulin polymers, arrest cell cycle progression and lead to cell death [23,24,25]. Several of these agents were approved for cancer treatment more than 50 years ago and are still the second most used class of chemotherapeutics [23,26,27]. However, their utility is often limited by the primary or acquired resistance and side effects, which can be severe and irreversible. Approximately 30–40% of BC patients treated with conventional anticancer therapies develop chemotherapy-induced peripheral neurotoxicity (CIPN), a common and debilitating side effect that significantly reduces quality of life. CIPN remains one of the most challenging adverse effects of chemotherapy and a major limiting factor in the treatment of BC [28]. It is a persistent morbidity that affects approximately 20–30% of BC patients long into survivorship, often presenting as a chronic and highly pharmacoresistant condition [29,30,31,32]. CIPN can arise after a single treatment or result from the cumulative effects of multiple agents. Its onset may necessitate dose reductions or even early discontinuation of chemotherapy - both of which can significantly compromise treatment efficacy and patient survival. In the United States, there are approximately 4 million BC survivors, with nearly 1 million experiencing chronic, debilitating consequences of their treatment.

Reducing the toxicities associated with conventional treatments remains a critical objective in the development of novel therapies for BC patients. This study examines a novel, microtubule-targeting compound, *N*-[5-(3′-iodobenzoyl)-1*H*-benzimidazol-2-yl]carbamate (IBCar), which demonstrates strong potential to improve treatment outcomes in BC. The oral bioavailability of IBCar was determined in healthy mice [33]. Pharmacokinetic analysis revealed a monophasic whole-body clearance profile, with a biological half-life of approximately 4 h following a single oral dose. The mean residence time was estimated at approximately 9 h. Brain uptake of IBCar at 1 h after oral administration was over 3000 times higher than previously reported for structurally related benzimidazole carbamates [34].

The primary objective of the current study was to evaluate the cytotoxicity, in vivo efficacy and safety of orally administered IBCar in BC cell lines and murine BC models. Our results strongly support the hypothesis that IBCar is a potent, orally bioavailable agent with significant therapeutic efficacy across multiple in vitro and in vivo models of breast cancer. Additionally, our findings indicate a notable resistance of normal cells to IBCar, suggesting an improved safety profile.

## 2. Materials and Methods

### 2.1. Cell Lines

Human breast cancer cell lines BT-549, HCC70, MDA-MB-175-VII, MDA-MB-231, MDA-MB-361, and MDA-MB-468 were purchased from American Type Culture Collection (ATCC, Manassas, VA, USA) and maintained in HyClone^TM^ Dulbecco’s Modified Eagle’s Medium (DMEM) containing 4500 mg/L glucose, 4.0 mM L-glutamine, and 110 mg/L sodium pyruvate, and supplemented with 10% fetal bovine serum (FBS; Cytiva, Logan, UT, USA). MCF-10A, a human epithelial cell line isolated from the fibrocystic breast, was also purchased from ATCC. Normal breast cells 76N, a human cell line derived from the mammary epithelial cells immortalized by human telomerase [35] was kindly contributed by Dr. Vimla Band (University of Nebraska Medical Center, Omaha, NE, USA). MCF-10A and 76N were maintained in Mammary Epithelial Growth Medium (MEBM) supplemented with Bullet Kit from Lonza Bioscience (Morrisville, NC, USA). Trypsin-EDTA (0.25%), Trypsin Neutralizing Solution, and sterile cell culture grade DMSO were obtained from VWR (Radnor, PA, USA). Non-enzymatic cell dissociation solution in phosphate buffered saline (PBS) without calcium and magnesium was purchased from Sigma-Aldrich (St. Louis, MO, USA). Cell proliferation was measured using the colorimetric MTS Assay Kit (Abcam, Boston, MA, USA). Cell line characteristics and abbreviations of cell line names are summarized in Table S3.

### 2.2. Chemicals, Reagents, and Antibodies

*N*-[5-(3′-iodobenzoyl)-1*H*-benzimidazol-2-yl]carbamate (IBCar), was synthesized, purified, and characterized as described previously [33,36,37]. Necrostatin-1 and vincristine were purchased from Selleck Chemicals (Houston, TX, USA). Antibodies and their sources are listed in Supplementary Materials (Table S1). Gel electrophoresis, Western blotting supplies, and their sources are listed in Supplementary Materials (Table S2). Assay and fluorescent dyes details and sources are listed in Supplementary Materials.

### 2.3. Computational Methods

The crystal structure of human tubulin was downloaded from the Protein Data Bank (PDB ID: 6BRY, resolution: 2.70 Å) [38]. Water molecules, ions, stathmin-4, and tubulin tyrosine ligase were removed from the downloaded structure. Crystallographic disorders and unfilled valence atoms were repaired using AutoDock Tools 1.5.7 [39]. Polar hydrogens and Kolmann charges were added. The entire protein was designated rigid in the PDBQT file. IBCar in the PDB format was prepared using online software [39,40]. The AD4 atom types were assigned, polar hydrogens added, bond order fixed, bonds renumbered, Gasteiger charges added, and file saved in the PDBQT format. A three-dimensional user-specified grid: 60 x-points × 60 y-points × 60 z-points grid with a spacing of 0.5 Å was centered on the αβ tubulin dimer. Affinity maps were calculated using AutoGrid 4.2.6. The Lamarckian genetic algorithm with a maximum of 2,500,000 energy evaluations was implemented in AutoDock4 [40]. AutoDock performed cluster analyses of the different docked conformations detecting the minimum energy in each run and selecting the 10 best-scoring solutions. Rmsd (root mean square deviation) cluster analysis was performed using only ligand atoms and outputting structurally similar clusters ranked in order of increasing energy (Autodock rmsd Table in Supplementary Materials). Seven distinct conformational clusters were found out of 10 runs using the rmsd-tolerance of 2.0 Å. Similar docking protocols were followed for colchicine and mebendazole to compare their binding energies and inhibition constants to IBCar (Table 1). For comparison, docking parameters of three regioisomers of IBCar (ortho, meta, and para) located in the colchicine binding site of the αβ tubulin dimer determined using the Schrödinger Suite (Schrödinger, New York, NY, USA) are shown in Figure S1.

### 2.4. Cell Proliferation, GI_50_ Determination, and Cytotoxicity Assays

Details of doubling time determination (T_D_s), cellular metabolic activity using the MTS assay, GI_50_ determination, clonogenic assays, and other methods to determine cell viability are provided in Supplementary Materials. In each methods, data analyses involved the calculation of surviving fractions (SF) as the fraction of viable cells or colonies relative to DMSO-treated control. SFs were then plotted as a function of concentration to determine half maximal growth inhibitory concentrations of IBCar (GI_50_). The nonlinear regression functions of GraphPad Prism 9 were used to determine GI_50_ and standard deviations (std dev).

### 2.5. Protein Extraction and Western Blotting

Cells grown to 70–80% confluence were incubated with IBCar (0.5 µM, 1 µM) for 24 h and 48 h. Spent medium was collected, cell monolayers washed twice with PBS. Cell lysates were prepared using commercial lysis buffers supplemented with protease and phosphatase inhibitors. Protein concentration in cell lysates was measured using Micro BCA^TM^ protein assay kit (Thermo Fisher Scientific, Waltham, MA, USA). For gel electrophoresis, cell lysate aliquots were added to the 2× sample buffer. Protein samples were denatured at 95 °C for 5 min, cooled, and loaded onto gels at 50–100 μg total protein per well. Gels electrophoresis and Western blotting were conducted using established protocols and are detailed in Supplementary Materials. Western blot protein band intensities corrected for the total protein load (GAPDH) are shown in Figure S2 (Supplementary Materials). Images of uncropped Western blots are also included in Supplementary Materials.

### 2.6. Effects of IBCar on the Integrity of Microtubules

Cells grown in T25 flasks to 70–80% confluence were incubated with IBCar (0.1 µM, 0.5 µM, 1 µM) for 1 h, 24 h, and 48 h. Medium was removed and monolayers washed with PBS. Stock solution of Tubulin Tracker^TM^ Deep Red (1000× in anhydrous DMSO) was diluted to 1× solution in the Invitrogen^TM^ Live Cell Imaging buffer (Thermo Fisher Scientific, Waltham, MA, USA) and added to monolayers. Cells were incubated for 30 min at 37 °C (5% CO_2_). One drop nucleus staining reagent (NucBlue^TM^ Live; Thermo Fisher Scientific, Waltham, MA, USA) was added and cell were incubated for additional 20 min at rt. Probenecid was diluted in Live Cell Imaging solution and used to wash out (3×) extracellular dyes from cells. Microtubules were imaged using FLoid^TM^ Cell Imaging Station λ_ex_ = 652 nm; λ_em_ = 669 nm (Thermo Fisher Scientific, Waltham, MA, USA).

### 2.7. Cell Cycle Analyses

Changes in the cell cycle phases were evaluated in BC cells and cells derived from normal breast tissue after treatment with various concentrations of IBCar for 24 h and 48 h. Untreated control cells were grown in medium containing DMSO. Cells were harvested using non-enzymatic dissociation buffer; washed twice with PBS and centrifuged at 1500 rpm for 10 min at 4 °C. Cell pellets were resuspended in ice-cold 70% ethanol and gently vortexed to obtain monodispersed cell suspensions. Cell suspensions were stored at −20 °C until all samples were ready for the flow cytometry analyses. Ethanol-fixed cells were centrifuged, ethanol decanted, and cells washed one time with PBS. The resultant cell pellets were resuspended, ~1 × 10^6^–5 × 10^6^ cells/mL, in the Telford reagent. Cell suspensions were kept in the dark at room temperature for 2 h. Stained cell suspensions were transferred to the flow cytometer and cell-associated fluorescence was measured.

### 2.8. Caspases Monitoring, Apoptosis, and Riptosome Analyses

#### 2.8.1. Caspases and Apoptosis

Two methods were used to evaluate caspase-3 (Cas-3) and caspase-7 (Cas-7) in BC cells: Western blot (described above) and cell imaging. For imaging, cells were grown in T25 flasks to 60–70% confluence and incubated with IBCar (0.1 µM, 0.5 µM, 1 µM) for 1 h, 3 h, 24 h, and 48 h. CellEvent^TM^ Caspase-3/7 Green Detection Reagent (Thermo Fisher Scientific, Waltham, MA, USA) was used to monitor Cas-3/7 activation using live cell fluorescence imaging. NucBlue^TM^ (Thermo Fisher Scientific, Waltham, MA, USA) was used to stain nuclei. Cells were imaged using the FLoid^TM^ Cell Imaging Station with filters set for FITC (λ_ex_ = 502 nm; λ_em_ = 530 nm).

#### 2.8.2. Riptosome Evaluation

The protein components of the riptosome were analyzed using Western blots. The role of RIP1 was evaluated using necrostatin-1, RIP1-targeted inhibitor. Cells were plated in wells of 96-well plates at predetermined optimal densities for each cell line and allowed to attach for 24 h. One set of wells was treated with necrostatin-1 at concentrations of 1, 10, 50, and 100 μM. The second set of wells on the same plate was treated with 0.1 μM IBCar concurrently with the indicated above concentrations of necrostatin-1. Cells were incubated with the reagents for 48 h. Medium was aspirated from wells, monolayer washed 1× PBS, and the LIVE/DEAD^TM^ Cell Imaging Kit 488/570 was used to determine cell survival as described above. A separate set of plates was also analyzed using the MTS assay to corroborate findings.

### 2.9. Evaluation of Endoplasmic Reticulum Stress and Mitochondrial Membrane Potential

#### 2.9.1. Endoplasmic Reticulum Stress

Analyses of several ER stress proteins were conducted using the human HSP Array C1 (RayBiotech Life, Inc., Peachtree Corners, GA, USA) and Western blotting. Lysates were prepared and Western blots performed as outlined above. The HSP array analysis was conducted according to the vendors protocol with the total protein load adjusted to 500 μg per membrane.

#### 2.9.2. Mitochondrial Membrane Potential

The Ion Vital-MitoVolt assay (Ion Biosciences, San Marcos, TX, USA) was used to determine the mitochondrial membrane potential using the protocol provided by the vendor. BC cells were plated into T25 flasks, allowed to recover and grow for 48 h, fresh medium added, and cells were either not treated (DMSO) or treated with 0.1 µM and 1 µM IBCar for 2 h, 24 h, and 48 h. The JC-10 dissolved in the dye loading buffer was added directly to cells at 1:2 (*v*/*v*) ratio. Cells were incubated for 60 min protected from light. The masking buffer was added directly to cells, and the cells imaged using the FLoid^TM^ Cell Imaging Station (λ_ex_ = 490 nm, 540 nm; λ_em_ = 525 nm, 590 nm). MitoBrilliant^TM^ Live 646 (Tocris, Bio-Techne, Minneapolis, MN, USA) was used to corroborate MitoVolt data. The dye solution diluted in growth medium was added to live cells. Cells were incubated for 60 min at 37 °C; washed 1× PBS; fresh medium was added, and mitochondria imaged (λ_ex_ = 648 nm; λ_em_ = 662 nm).

### 2.10. Mouse Tumor Models and Treatments

Animal experiments were conducted in compliance with the University of Nebraska Medical Center institutional guidelines using protocols approved by the Institutional Animal Care and Use Committee. Mice were housed in a pathogen-free facilities at controlled temperature and humidity with a 12-h light–dark cycle. Food and water were provided ad libitum. Mice were examined daily. Their body weights were measured three times a week. Throughout the course of the study, tumor dimensions were measured twice per week using Scienceware^®^ Digi-Max^TM^ slide caliper (Sigma-Aldrich), and the tumor volume was calculated: *volume* = [*π* × (*width*)^2^ × (*length*)]/6.

#### 2.10.1. MMTV-PyMT Transgenic Breast Cancer Mouse Model

To evaluate the effects of IBCar on the mammary gland tumor formation and lung metastases, FVB/N transgenic mice expressing the PyMT oncogene under the control of the MMTV LTR promoter/enhancer were used [41]. Female, 4-weeks old FVB/N-Tg(MMTV-PyVT)634Mul/J mice were purchased from the Jackson Laboratories (strain 002374; Bar Harbor, ME, USA). Female mice develop palpable mammary tumors with a mean latency of 53 days of age [42]. At 40 days of age, mice were randomly assigned into two groups. NT group (n = 6): control mice (25 µL of PBS containing 5% ethanol); IBCar group (n = 6): oral therapy mice (PO dose of IBCar 10 mg/kg bw in 25 µL of PBS containing 5% ethanol). Mice were palpated daily to check the development of mammary tumors. IBCar and vehicle doses were administered every other day for 31 days with weekend breaks. Mice in the NT group were euthanized consecutively as their total volume of mammary gland tumors approached ~2 cm^3^. IBCar-treated mice were euthanized at the age of 88 days. Mammary tumor tissue, lungs, and kidneys were collected and weighed. Blood was analyzed using the VetScan HM5 hematology system (Zoetis, Parsippany, NJ, USA). Aliquots of blood were processed for serum. Blood chemistry analyses were performed using the VetScan^®^ VS2 chemistry analyzer (Zoetis). Frozen tumors and lungs were minced and transferred into a volume of ice-cold lysis buffer equivalent to two tumor weights (20 mM Tris, pH 7.5, 150 mM NaCl, 1 mM EDTA, 1 mM EGTA, 1% Triton X-100, 2.5 mM Na_4_O_7_P_2_, 1 mM β-glycerol-phosphate, 1 mM Na_3_VO_4_, 1 µg/mL leupeptin, supplemented with 1 mM PMSF). Minced tumor fragments were placed in a tube on ice and processed first with the tissue tearor (MIDSCI, St. Louis, MO, USA) then sonicated using a Vibra Cell Model VC 375 ultrasonic processor (Sonics&Materials, Inc., Danbury, CT, USA) for 14 s with a 14-s break between sonications for a total of 4 min at a 40% duty cycle. Homogenates were centrifuged at 14,000× *g* for 10 min at 4 °C. Supernatants were collected, aliquoted and stored at −80 °C. Prior to Western blot analyses of estrogen (ER) and progesterone (PR) receptors the total protein concentration was determined using the micro-BCA assay (Thermo Fisher Scientific, Waltham, MA, USA).

#### 2.10.2. Human Xenograft Mouse Models

Xenografts were established in immunodeficient outbred nude J:NU female mice homozygous for *Foxn1^nu^*/*Foxn1^nu^* (The Jackson Laboratories, Bar Harbor, ME, USA). BC cells in 0.2 mL VitroGel^®^ Hydrogel Matrix, 5 × 10^6^ per mouse, were implanted on the left flank. Mice received the implant of BC cells at the age of 38 days. Three days after the cell implant, mice were randomly divided into two groups: NT control group (n = 9): mice treated PO with 25 µL of PBS containing 5% ethanol; IBCar therapy group (n = 10): mice treated with PO doses of IBCar 10 mg/kg bw in 25 µL of PBS containing 5% ethanol. Nine days after BC cell implants tumors reached an average volume of ~120 mm^3^ and treatments commenced. PBS and IBCar were given approximately every 2 days for 26 days with weekend breaks. Mice were observed for an additional 20 days. The experiment was terminated when one xenograft in the NT group developed a small ulcer (as per the UNMC IACUC policy). Xenografts, lungs, liver, spleen and kidneys were collected, weighed, and macroscopically evaluated. Analyses of blood hematology and serum chemistry were performed as described above.

### 2.11. Statistical Analyses

Statistical analyses were performed with GraphPad Prism 10 software (GraphPad Software, Boston, MA, USA). Table S4 in Supplementary Materials provides statistical details for the evaluation of IBCar’s cytotoxicity. Tables S5 and S8–S11 in Supplementary Materials provide details of statistical analyses for the in vivo studies. Data are reported as mean ± standard error or ±standard deviation, and statistical significance is indicated (*p* values). The multiwell assays data were analyzed using nested one-way AVOVA with Tukey’s multiple comparisons test. For the GI_50_ cytotoxicity determination, responses to IBCar were normalized to the DMSO-treated controls (surviving fractions; SF), plotted on a semi-log scale, and analyzed using the non-linear regression functions in GraphPad Prism. GI_50_s were derived for each cell line from n = 4–6 technical and two biological (passage number) replicates. GI_50_s values determined in the nonlinear regression were compared using the ordinary ANOVA with Dunnett’s multiple comparisons test (Table S4). Clonogenic assay data were normalized to DMSO-treated controls to calculate SF and analyzed using one-way ANOVA with Tukey’s pairwise comparisons. Unpaired *t*-test with Welch’s correction was used to assess differences in SFs between cell lines. Tumor growth curves were analyzed using the GraphPad exponential (Malthusian) growth function assuming a constant doubling time. Weights of extirpated xenografts, mammary tumors, and tumor growth curves were analyzed using unpaired *t*-test with Welch’s correction.

## 3. Results

### 3.1. Molecular Docking Studies

**Figure 1:**
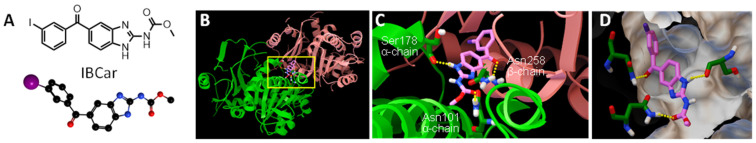
(**A**). Chemical structure of IBCar. (**B**). Molecular docking analyses of IBCar interactions with α- and β-chains of tubulin. (**C**). Close-up of IBCar in its binding site. (**D**). Molecular surface of the IBCar binding pocket in the β-chain. Hydrogen bonds with Asn101 (α-chain), Ser178 (α-chain), and Asn258 (β-chain) are shown as yellow dots.

Molecular docking studies were conducted prior to laboratory experiments to estimate the potential of IBCar as a microtubule-binding agent, in the context of compounds with similar activities toward microtubules. The interaction between IBCar and the α- and β-chains of tubulin was investigated using AutoDock. Figure 1 shows a graphical depiction of these interactions. The binding free energy was estimated at −11.44 kcal/mol (Table 2), with an inhibition constant (K_i_) of 4.08 nM at 298.15 K. The final intermolecular energy was estimated at −12.64 kcal/mol, with the electrostatic energy of 0.00 kcal/mol; a final total internal energy of −0.76 kcal/mol; a torsional free energy of +1.19 kcal/mol; and an unbound system’s energy of −0.76 kcal/mol. The top-ranked docking pose revealed three hydrogen bonds between IBCar and tubulin: two with residues Asn101 and Ser178 in the α-chain, and one with Asn258 in the β-chain.

The molecular docking parameters of IBCar were compared to mebendazole and colchicine. Mebendazole, a structural analog of IBCar, is currently being repurposed for the treatment of several cancers, including breast cancer. Our previous study established cytotoxic effects of mebendazole in several BC cell lines [43]. The half-maximal effective concentrations (EC_50_) of mebendazole ranged from approximately 0.2 µM to 0.7 µM, confirming the predicted superiority of IBCar. Colchicine, an anti-microtubule agent, inhibits the polymerization of tubulin into microtubules. Its binding site, located at the interface between α- and β-tubulin subunits, is similar to that of IBCar. Specifically, colchicine interacts with Thr179 in the α-chain and Ala180 and Val181 in the β-chain. Given these similarities, we considered it important to compare IBCar with these two agents. The predicted K_i_ of 4.08 nM for IBCar is substantially lower than that of colchicine and mebendazole (Table 1).

### 3.2. Selective Cytotoxic Effects of IBCar in Normal Versus Breast Cancer Cells

The characteristics of the cell lines used in this project are listed in Table S3. Cell doubling times (T_D_s) were determined prior to conducting the cytotoxicity tests. The effects of IBCar on both BC and normal cells were assessed and validated using three independent methods: the MTS assay to measure cytotoxicity at early time points following exposure to IBCar; the clonogenic assay, which is the gold standard for survival analysis of cells capable of producing colonies; and SYTOX^TM^ Green dead cell staining combined with Hoechst 33,342 staining to count total nuclei, validating the MTS assay results in cells that do not form colonies. In all assays, cells were treated with various concentrations of IBCar, alongside controls grown in medium containing DMSO. The efficacy of IBCar was also confirmed through the NCI-60 Human Tumor Cell Lines Screen conducted by the NCI DTP (Table S8).

**Figure 2:**
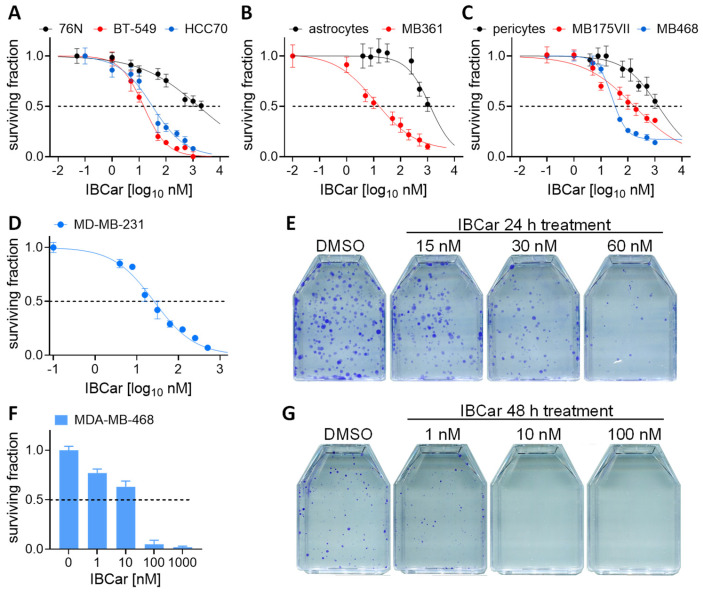
Cytotoxicity of IBCar in normal cells and BC cells. (**A**–**C**) Cell survival after 48 h treatment with IBCar determined using the MTS assay. (**A**) Comparison of IBCar cytotoxicity in BT549 and HCC70 cells derived from the primary tumor with normal breast 76N cells. (**B**) Comparison of IBCar cytotoxicity in MB361 cells derived from brain metastases with human astrocytes. (**C**) Comparison of IBCar cytotoxicity in MB175 and MB468 cells derived from pleural effusions with human pericytes. (**D**–**G**) Survival of BC cells treated with IBCar determined using the clonogenic assay. (**D**) Surviving fraction of MB231 cells after 24 h with IBCar. (**E**) Colonies of MB231 cells: controls (DMSO) or treated with IBCar for 24 h. (**F**) Surviving fraction of MB468 cells after 48 h with IBCar. (**G**) Colonies of MB468 cells: controls (DMSO) or treated with IBCar for 48 h.

Cell metabolic activities were measured after 24 h, 48 h, and 72 h of IBCar treatment (Table 2). A similar experimental design was employed to determine GI50 values for normal breast epithelial cells (76N), astrocytes, and pericytes. In the clonogenic assay, cells were treated with IBCar for 24 h or 48 h, followed by up to 21 days of culture in IBCar-free medium. Figure 2 illustrates the responses of BC and normal breast cells to IBCar. The MTS assay results in BC cells were corroborated by data obtained from the clonogenic assays (Figure 2D–G).

Comparisons were made between BC cells and normal cells inherent to the environments from which the tumor cells originated. The responses of 76N normal breast cells to IBCar were compared to two primary TNBC BC cell lines with mutated *TP53* (Figure 2A). The survival of astrocytes was compared to MB361 cells, which were derived from a brain metastatic site (Figure 2B). Responses of pericytes to IBCar were compared to BC cells derived from pleural effusion (Figure 2C). Pericytes served as a model for a normal microenvironment that, when damaged by chemotherapy, can acquire pro-metastatic characteristics [44]. Several studies also suggest that the loss of pericytes contributes to increased metastasis in mice [45], indicating that such a loss may create local microenvironments conducive to promoting metastasis [46,47]. By employing several independent methods, we demonstrated that BC cells are effectively eradicated at concentrations < 25 nM, whereas normal cells exhibit significant resistance to IBCar.

### 3.3. Cell Cycle Effect of IBCar

**Figure 3:**
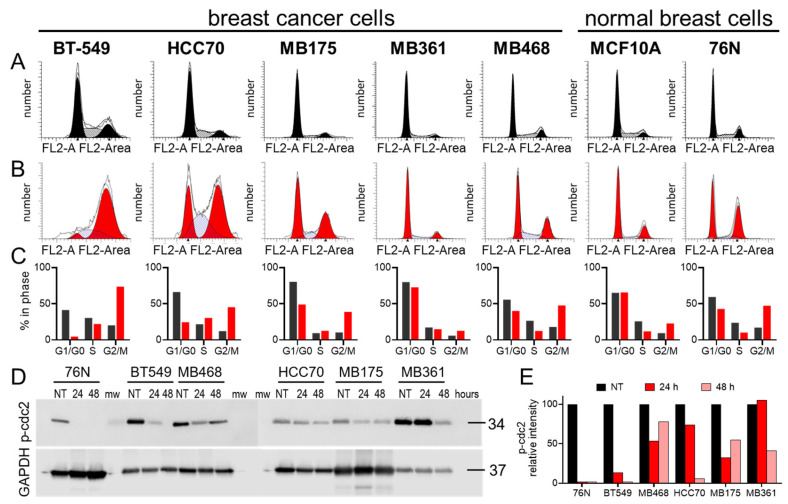
Cell cycle analyses of normal breast and breast cancer cells in response to IBCar treatment. (**A**). Untreated control cells (DMSO). (**B**). Cells treated with 500 nM IBCar for 48 h. (**C**). Summary of the cell cycle phase changes. Black bars = DMSO control; red bars = IBCar -treated cells. (**D**). Western blot analyses of cdc2 dephosphorylation at Tyr15 resulting in the cdc2 activation during progression into mitosis; NT denotes untreated cells; 24, 48 denote cells treated with IBCar for 24 h or 48 h, respectively; mw marks lanes with molecular weight markers. (**E**). Relative intensity of p(Tyr15)-cdc2 bands normalized to GAPDH band intensities and p(Tyr15)-cdc2 in untreated cells (100%).

Cell cycle analyses using the Telford method with propidium iodine staining showed that IBCar treatment induced cell cycle arrest at the G2/M phase (Figure 3A–C). Of the seven cell lines tested, TNBC BT549 and HCC70 cells underwent the most pronounced shift to the G2/M phase. Both cell lines were derived from primary tumors harboring *TP53* hotspot mutations associated with gain-of-function phenotypes [48,49]. The G2/M arrest was confirmed using the Western blot of p(Tyr15)-cdc2 with anti-pcdc2 rabbit antibodies in conjunction with the analyses of H3 histone phosphorylation at Ser-10 (Figure S5). The critical regulatory step for cdc2 during progression into mitosis is its dephosphorylation at Tyr15 [50]. It is evident (Figure 3D) that the entry of all tested cells into mitosis is regulated by the cdc2 kinase activation regardless of the *TP53* status. Figure 3E shows the declining levels of p-cdc2 normalized to the intensity of GAPDH and untreated controls protein band intensities. We can tentatively propose that the normal cells resistance to IBCar is not related to the *TP53* status. 

### 3.4. Cell Death Mechanisms

The objective was to determine whether the cell death mechanisms induced by IBCar treatment differ between BC cells and normal breast cells, and to explore any underlying dissimilarities. Live cell imaging and Western blot analyses were used to assess cellular death events.

#### 3.4.1. Caspase-3 and Caspase-7 Activation

**Figure 4:**
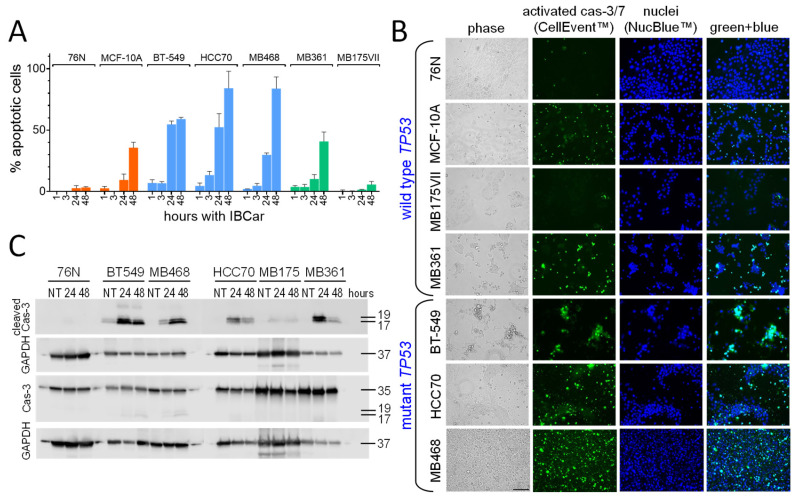
Determination of IBCar effects on apoptosis. (**A**). Expansion of the apoptotic cell population during the IBCar treatment of normal breast cells (orange bars), m*TP53* BC cells (blue bars), and wt*TP53* BC cells (green bars). Percentages are the ratio of cells expressing activated Cas-3/7 to all cells in the population. (**B**). Evaluation of caspase-3/7 activation in normal breast and BC cells treated with IBCar for 48 h. Apoptotic cells are green. All cell except MB468 (0.5 µM IBCar) were treated with 1 µM IBCar. Black bar = 100 µm. (**C**). Expression of caspase-3 and cleaved caspase-3 in normal breast cells and BC cells; NT = untreated cells; 24, 48 = cells treated with 0.5 µM IBCar for 24 h or 48 h, respectively.

Apoptotic responses of BC and normal breast cells to IBCar were evaluated using caspase-3/7 (Cas-3/7) activation assays (Figure 4A,B), as well as Western blot analyses of total and cleaved Cas-3 (Figure 4C), and PARP (Figure S4). Apoptotic cells were visualized using CellEvent^TM^ Cas-3/7 Green, a reagent suitable for longitudinal assessment of Cas-3/7 activation in monolayer cultures over 72 h without disturbing the cells. The reagent is intrinsically non-fluorescent; upon activation of Cas-3/7 in cells, the DEVD peptide is cleaved, binds to DNA, and generates a bright green fluorescence signal (Figure 4B).

All BC cell lines except MB175 showed Cas-3/7 activation following IBCar treatment. MB175 cells exhibited minimal apoptosis, and only after prolonged IBCar exposure (>48 h). However, MB175 cells displayed significant nuclear fragmentation in over 25% of nuclei as early as 24 h post-treatment, suggesting mitotic catastrophe as a potential mechanism of cell death (Figure S9). In several BC cell lines, Cas-3/7 activation was detectable as early as 1 h after exposure to IBCar (Figure 4A). In contrast, in 76N normal breast cells, the apoptotic population remained undetectable even after prolonged exposure to IBCar. Baseline Cas-3/7 activity was minimal across all tested cell lines (Figure S10). Although the size of the apoptotic fraction varied between cell lines (Figure 4A), it consistently increased over time in each case. However, a clear concentration-dependent response to IBCar was not apparent. For example, MB468 and HCC70 cells exhibited nearly identical levels of apoptosis at both 0.1 µM and 0.5 µM concentrations (Figure S11).

Cas-3/7 imaging data were substantiated using Western blot analyses of lysates from control cells (DMSO) and cells treated with IBCar for 24 h and 48 h (Figure 4C). BT549 cells showed high levels of Cas-3 fragments p17 and p19 after 24 h and 48 h with IBCar. This coincided with the large apoptotic fraction observed in the Cas-3/7 imaging studies. High levels of cleaved Cas-3 in MB468 after 48 h with IBCar also paralleled the observation from the Cas-3/7 imaging studies. The p17 and p19 fragments of cleaved Cas-3 were not detected in lysates from 76N and MB175 cells treated with IBCar, supporting the observed lack of Cas-3/7 activation in these cell lines.

#### 3.4.2. Caspase-8

Procaspase-8 (Cas-8) activity correlates with Cas-8 prodomain cleavage at Asp374 between the catalytic subdomains, which generates fragments p43 and p41. These fragments typically emerge first after activation [51,52,53]. Fragments p43 and p41 are intermediate products in the Cas-8 maturation process and are responsible for most of the Cas-8 activity in cells [53]. The cleavage of Cas-8 also produces p18 and p10 fragments.

**Figure 5:**
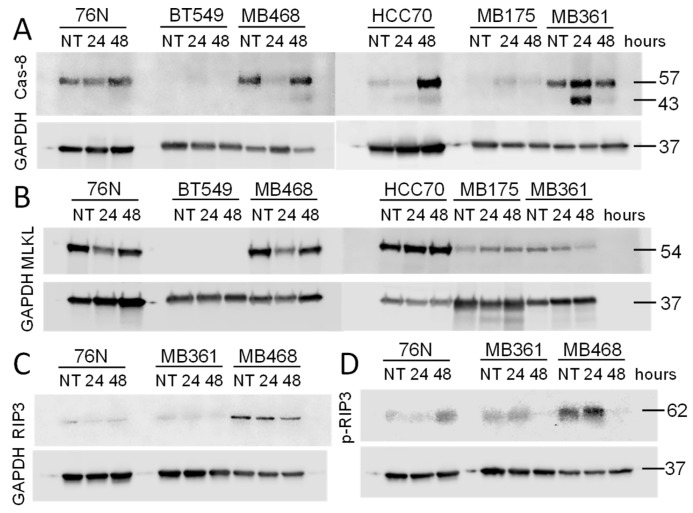
Expression of Cas-8 (**A**), MLKL (**B**), RIP3 (**C**), and pRIP3 (**D**) in normal breast cells and BC cells treated with IBCar; NT = untreated cells; 24, 48 = cells treated with IBCar for 24 h or 48 h, respectively.

**Figure 6:**
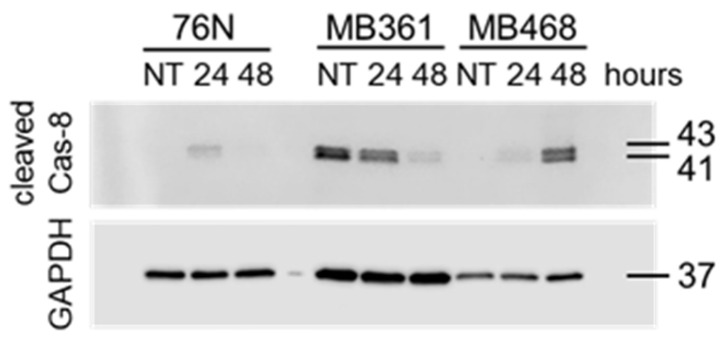
Expression of cleaved Cas-8 in normal and BC cells. Untreated cells (NT) were grown in DMSO-containing medium. Cells were lysed after 24 h and 48 h with 0.5 µM IBCar.

Untreated normal breast cells (76N), as well as BC cell lines MB361 and MB468, express full-length Cas-8 (Figure 5A). In contrast, Cas-8 expression was nearly undetectable in untreated BT549, HCC70, and MB175 cells (Figure 5 and Figure S6). Upon IBCar treatment, full-length Cas-8 expression was upregulated in HCC70 cells. Although the 18 kDa and 10 kDa Cas-8 cleavage fragments were not detected, likely due to their short half-life of approximately 7 min [54], we observed the presence of the 41 kDa and 43 kDa fragments (Figure 6). However, only in IBCar-treated MB361 and MB468 cells, which also had high levels of cleaved Cas-3 and activated Cas-3/7. MB468 lysates also had a weak 18-kDa band after 48 h treatment with IBCar. During apoptosis, Cas-8 substrates, such as downstream effector caspases Cas-3 and Cas-7, are cleaved and activated by Cas-8 p43 and p41 intermediates [55]. The notable lack of Cas-8 p41 and p43 fragments in 76N cells, despite the prominent presence of the full-length protein, confirms that apoptosis is not an active cell death mechanism in this cell line. Moreover, these data suggest that dissimilarities in Cas-8 expression and maturation may underline the differences in cellular responses to IBCar, i.e., the relative resistance of normal breast cells to IBCar and its lethality in BC cells.

### 3.5. Riptosome

The receptor-interacting protein (RIP) family of serine/threonine kinases are important regulators of cellular stress, capable of activating both pro-survival and pro-apoptotic pathways [56]. These processes are tightly regulated by a balance between RIP1 activation, fragmentation, and degradation [57,58,59,60]. For these reasons, we investigated the potential role of RIP in mediating the observed resistance of normal cells to IBCar.

All BC and normal breast cell lines tested to date, except MB468, appear to be RIP3-deficient (Figure 5C). One probable reason is the suppression of necroptosis by epigenetic silencing of RIP3 [61]. Although 76N normal breast cells express full-length Cas-8, cleaved Cas-8 is not generated in response to IBCar treatment (Figure 5 and Figure 6). High levels of Cas-8 can promote apoptosis by enabling its self-cleavage into active forms. Conversely, heterodimerization of Cas-8 inhibits apoptotic cell death, leading to increased levels of active RIP1, which can phosphorylate RIP3, resulting in MLKL-dependent necroptosis [62]. All tested cell lines, except BT549, express varying levels of MLKL (Figure 5B), however, we were unable to detect S345-phosphorylated MLKL in any of them consistent with RIP3-deficiency in tested BC cell lines.

**Figure 7:**
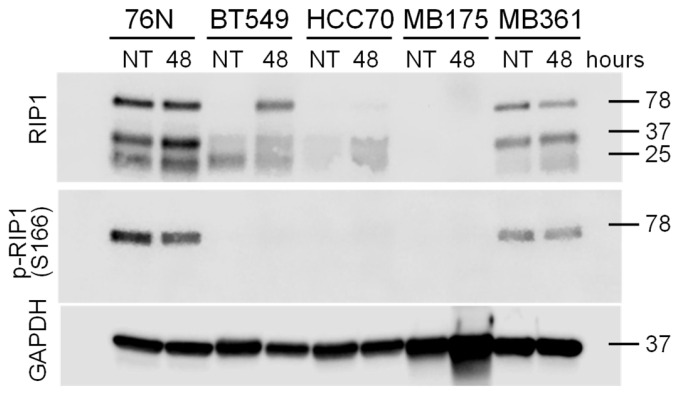
Expression of RIP1 and its Ser166 phosphorylated derivative in normal breast and BC cells. Untreated cells (NT) were grown in DMSO-containing medium. Cells were treated with 0.5 µM IBCar for 48 h.

Untreated cells 76N and MB361 have detectable levels of RIP1 (Figure 7). RIP1 expression in BT549 cells is induced upon IBCar treatment. 76N and MB361 cells, untreated controls as well as IBCar treated cells, also express RIP1 phosphorylated at Ser166, an autophosphorylation site in the RIP1 kinase domain. In 76N cells, RIP1 is constitutively cleaved at the N-terminus, generating p25 and p37 fragments. These fragments were detected using antibodies targeting an epitope centered around Leu190 in the kinase domain (Figure 7). Even though RIP1 cleavage is primarily mediated by Cas-8, the process can be executed by Cas-3 or Cas-6 in cell lacking Cas-8 [63]. The absence of activated Cas-3 can attenuate RIP1 proteolysis [64]. The RIP1 fragmentation at the N-terminus (kinase domain) suppresses the apoptotic and necrotic RIP1 activities and promotes cell survival [59], which appears to be the case for 76N cells. When activated Cas-8 is inhibited or absent, as observed in 76N cells (Figure 6), RIP1 exhibits a dual role, promoting either cell survival or necroptosis, depending on the cellular context [57]. The cleavage site of RIP1 appears to have a decisive role in determining the cellular fate. In 76N normal breast epithelial cells treated with IBCar, this mechanism favors pro-survival signaling, which correlates with the intrinsic presence of elevated levels of N-terminal RIP1 kinase domain fragments. Recent studies suggest that RIP1 inhibition can prevent necroptosis in the brain [65], and that both RIP1 and Cas-8 also contribute to cell-death-independent processes, including proper chromosome alignment during mitosis [60]. Vinblastine—which like IBCar depolymerizes microtubules—can amplify the expression of necrosome components RIP1, RIP3, and MLKL [66]. IBCar, despite its similar effects on microtubules, does not have any significant impact on the expression of these three proteins in examined BC cells. However, IBCar treatment increases the N-terminal fragmentation of RIP1 in 76N and MB361 cells (Figure 7).

**Figure 8:**
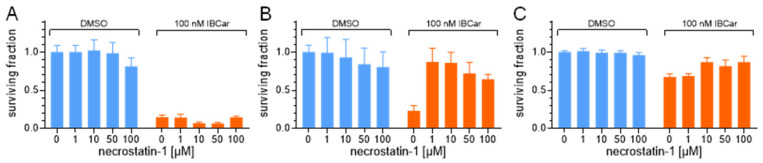
Effects of necrostatin-1 on survival of MB468 cells (**A**), MB361 (**B**) and 76N (**C**) cells. Control cells (n = 6; blue bars) were cultured with various concentration of necrostatin-1 in DMSO-containing medium. Experimental groups (n = 6; orange bars) were treated with the identical concentrations of necrostatin-1 and 100 nM IBCar.

Necrostatin-1 is widely used in various disease models to investigate the role of RIP1 in regulating cell survival, death, and inflammation [67]. It has been shown to alleviate peripheral nerve injury-induced pain by inhibiting the RIP1/RIP3 signaling pathway. Given its established function as a RIP1 inhibitor, we examined its effect on cell survival in response to IBCar treatment (Figure 8). Necrostatin-1 had no effect on the survival of IBCar-treated MB468 cells, consistent with the lack of RIP1 expression in these cells (Figure 8A). The results in MB468 cells are representative of findings in other RIP1-deficient breast cancer cell lines. Necrostatin-1, however, rescued RIP1-expressing MB361 cells from the cytotoxic effects of IBCar (Figure 8B). Its inhibitory effect was less pronounced in IBCar-treated 76N cells, suggesting that RIP1 cleavage within the kinase domain may independently contribute to the relative resistance of these normal cells to IBCar (Figure 8C). Our investigation to understand the full scope of RIP1’s role in mediating the response of both BC and normal cells to IBCar is ongoing.

### 3.6. Microtubule Integrity

**Figure 9:**
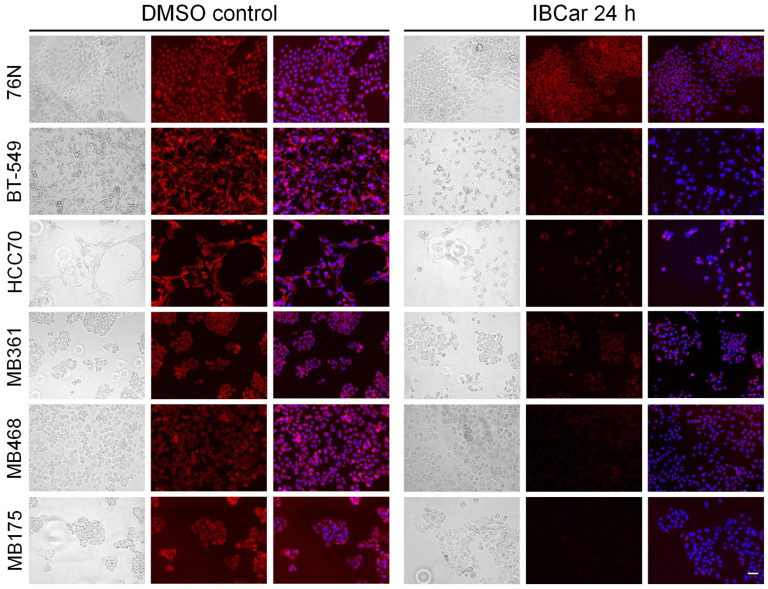
Evaluation of the microtubule integrity in 76N normal breast cells and several BC cell lines. Microtubules (red) are stained with Tubulin Tracker^TM^ Deep Red. Nuclei (blue) are stained with NucBlue^TM^ Live. Cells were either untreated (DMSO) or treated with 0.5 µM IBCar for 24 h. Phase contrast micrographs are included to show morphology. White bar in the bottom image = 100 µm.

During normal cellular functions, microtubules undergo polymerization and depolymerization of α- and β-tubulin heterodimers. IBCar binds to a site located between the α- and β-tubulin chains (Figure 1). Treatment with IBCar led to rapid and extensive microtubule depolymerization in all tested BC cell lines. In contrast, microtubule depolymerization in 76N normal breast cells was minimal. Figure 9 illustrates the status of microtubule structures in both BC and normal breast cells after 24 h of treatment with 0.5 µM IBCar.

**Figure 10:**
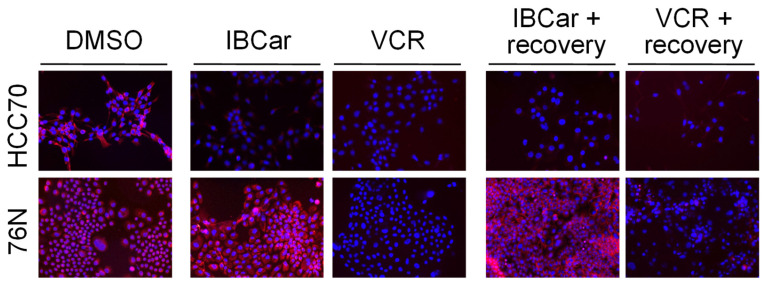
Evaluation of the microtubule integrity in breast cancer HCC70 cells and normal breast 76N cells after treatment with 0.5 µM IBCar for 48 h or 0.5 µM VCR for 24 h. Recovery: drug-containing medium was removed; cells washed with growth medium without drugs and cells allowed to grow for additional 72 h in fresh medium. Untreated control cells were grown in medium containing DMSO. Microtubules = red (Tubulin Tracker^TM^). Nuclei = blue (NucBlue^TM^).

The evaluation of microtubule integrity after IBCar treatment provided further evidence of the differing responses of normal breast cells and BC cells to IBCar. Data demonstrated reversibility of IBCar effects in normal 76N cells, which contrasts with the irreversible microtubule depolymerization by vincristine (VCR) in these cells (Figure 10). Treatment with VCR at the same concentration as IBCar, but for a shorter duration (24 h) followed by 72 h without the drug, did not result in any noticeable reconstruction of normal microtubule structures. Neither BC nor normal cells regained microtubule integrity after VCR treatment.

Microtubule depolymerization induced by IBCar in BC cells was permanent (Figure 10 and Figure S12–S15), whereas in normal 76N breast cells, only minor changes were observed, and these were transient. Partially depolymerized microtubules in 76N cells appear to have the capacity to recover their structure once IBCar is removed and the cells continue to grow in IBCar-free medium. The images in Figure 10 exemplify the data for all tested BC cell lines after 24 h of IBCar treatment, followed by 72 h of recovery in fresh medium. Of the HCC70 cells that survived IBCar treatment, none regained their microtubule structures, even when allowed to recover and grow in IBCar-free medium for 96 h. This represents a significant difference in cellular responses of BC and normal cells to IBCar versus VCR, which will be further explored in future studies.

### 3.7. Endoplasmic Reticulum Stress and Mitochondrial Membrane Potential

To gain a broader understanding of the differences between normal and BC cells’ responses to IBCar, we conducted further analyses to identify additional IBCar-induced stressors. Specifically, we focused on examining endoplasmic reticulum (ER) and mitochondrial stress responses.

#### 3.7.1. Endoplasmic Reticulum Stress Pathways Evaluation

**Figure 11:**
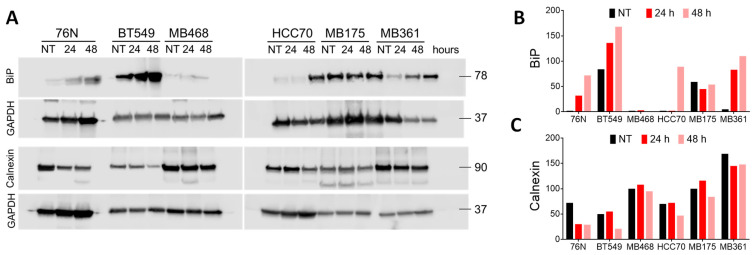
Expression of the endoplasmic reticulum sequential chaperones BiP and calnexin in untreated cells (NT) and cells treated with IBCar for 24 h and 48 h. (**A**). Western blots of cells lysates prepared from combined non-adherent and adherent cells. BiP (**B**) and calnexin (**C**) protein bands normalized to GAPDH levels.

The endoplasmic reticulum plays a critical role in protein folding and in maintaining its own homeostasis. When these functions are disrupted, ER stress signals are triggered, leading to either adaptive or apoptotic responses. The ER contains a pool of molecular chaperones, including BiP (GRP78) and calnexin, two transmembrane proteins that are synthesized on polysomes and translocated into the ER [68,69,70]. We analyzed BiP and calnexin expression in untreated and IBCar-treated cells using Western blotting. Cell lysates were prepared from combined fractions of non-adherent floating cells (which form when IBCar depolymerizes microtubules, although the cells are not yet dead) and non-enzymatically harvested adherent cells. Figure 11 summarizes our findings. All tested BC cell lines, with the exception of MB468 and MB175, showed a significant increase in BiP expression in response to IBCar treatment. Disruption of protein folding leads to increased synthesis of BiP to prevent protein aggregation and facilitate protein folding [71,72,73,74]. Previous studies have shown that BiP expression is upregulated by vinblastine, a compound that, like IBCar, disrupts microtubule integrity [74]. Our findings suggest that ER stress pathways represent an important mechanism of BC cells response to IBCar treatment. Untreated BT549 and MB175 cells exhibit intrinsically high levels of BiP. In contrast, normal breast epithelial 76N cells display only a weak BiP signal, which becomes detectable only when protein loading is increased nearly tenfold compared to other lysates.

Calnexin levels in BC cells remain largely unaffected by IBCar treatment. In contrast, normal breast epithelial 76N cells exhibit a nearly 250% reduction in calnexin levels (normalized to GAPDH) after 24 h of IBCar exposure (Figure 11A,C). Beyond its role in the unfolded protein response, calnexin also regulates mitochondrial energy production and stress response by modulating Ca^2+^ import into ER and facilitating Ca^2+^ transfer to mitochondria [75]. The observed decline in calnexin expression in IBCar-treated 76N cells may help preserve mitochondrial membrane potential (ΔΨ), thereby enhancing cell survival. This hypothesis is supported by our analysis of ΔΨ, which indicated that reduced calnexin levels contributed to the protective effect. The role of IBCar-induced microtubule destabilization in ΔΨ modulation is addressed in the following section.

**Figure 12:**
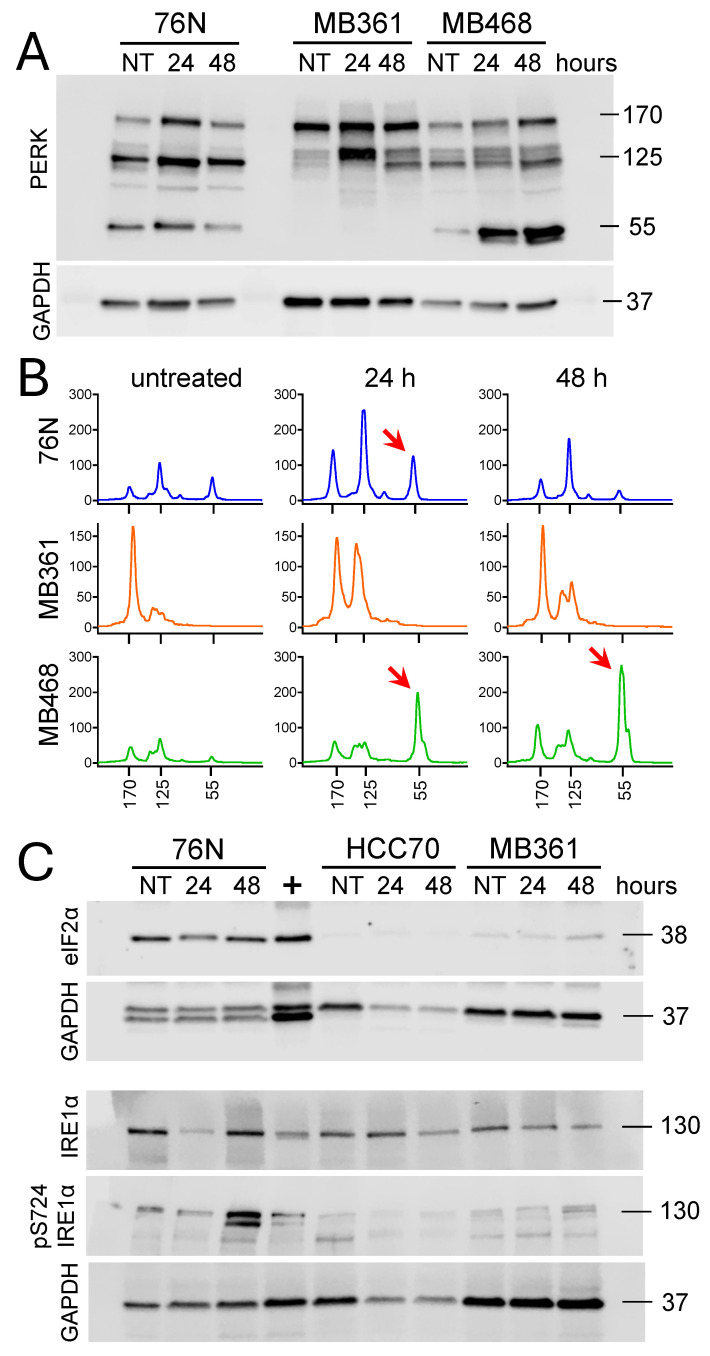
Expression of endoplasmic reticulum unfolded protein response sensors in untreated cells (NT) and cells treated with IBCar for 24 h and 48 h. (**A**). Western blot of PERK. (**B**). Analysis of changes in PERK expression. Red arrows indicate active PERK fragment. (**C**). Western blot of eIF2α, IRE1α, and phospho-Ser724-IRE1α. Positive control lysates are indicated by +.

To further investigate the role of IBCar as a cellular stressor, we analyzed the expression of protein kinase-like endoplasmic reticulum kinase (PERK, also known as EIF2αK3) and the transmembrane serine/threonine kinase inositol-requiring enzyme 1 alpha (IRE1α). These two proteins independently monitor the equilibrium between protein load and folding capacity and regulate key signal transduction pathways of the unfolded protein response [76,77,78]. Eukaryotic initiation factor 2α subunit (eIF2α) is a downstream effector of PERK [77,79,80]. We have analyzed the expression of these three proteins in untreated cells and cells exposed to IBCar for 24 h and 48 h (Figure 12). Data provide additional evidence in support of IBCar as the cellular ER stressor. ER stress increases the activity of PERK (Figure 12A,B). In 76N and MB361 cells, total PERK expression rises significantly after 24 h of IBCar treatment, followed by a marked decline at 48 h. Whereas, in MB468 cells, PERK activity continues to intensify in a time-dependent manner throughout the treatment period. Specifically, the expression of 55 kDa PERK protein kinase fragment increases ~10-fold and ~20-fold at 24 h and 48 with IBCar, respectively. Normal 76N cells double the expression of 55 kDa fragment after 24 h IBCar. However, at 48 h the expression of 55 kDa protein kinase fragment returns to basal levels observed in untreated 76N cells. MB361 cells do not produce 55 kDa PERK fragments. eIF2α is detectable exclusively in 76N normal breast cells (Figure 12C). Levels of IRE1α remain unchanged in MB361 and MB468 cells treated with IBCar. In contrast, IRE1α and p-Ser724-IRE1α are significantly upregulated in 76N cells treated with IBCar for 48 h suggesting that in this cell line two independent unfolded protein responses may be functional. The duration of PERK and IRE1α signaling can vary following disruptions in the protein folding processes. Sustained periods of PERK activities are detrimental to cell survival. The corresponding duration of IRE1α signaling is not [81,82]. Therefore, the transient PERK activity in 76N cells is suggestive of cytoprotective functions whereas the extended PERK activity in MB468 appears to contribute to BC cell death [81]. Extensive additional studies will be required to fully correlate the longitudinal interplay between these two proteins in IBCar-treated cells.

**Figure 13:**
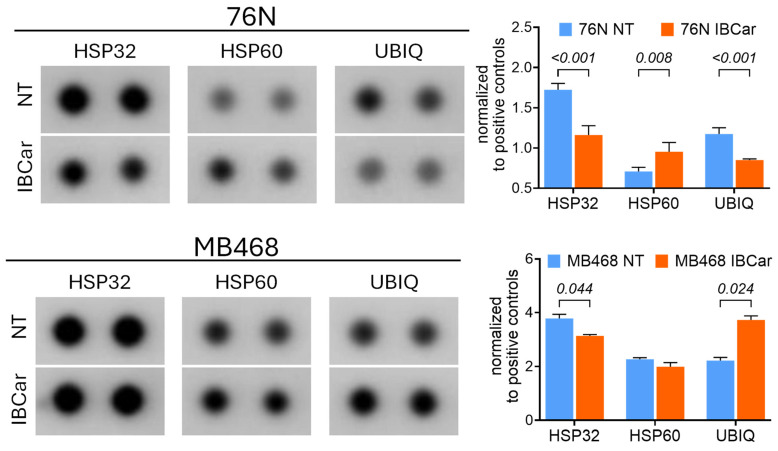
Human heat shock proteins expression in lysates from normal breast cells and BC cells either untreated (NT) and IBCar-treated (500 nM IBCar, 24 h). UBIQ = RPS27a.

Because mitochondrial proteins are also involved in the unfolded protein response, we analyzed the expression of six heat shock proteins. The data revealed differences between normal 76N cells and MB648 cells (Figure 13 and Figure S7). In normal breast cells, IBCar treatment decreases the expression of HSP32 and ubiquitin-ribosomal protein eS31 fusion protein (RPS27a) and upregulates HSP60. In MB468 cells, neither HSP32 nor HSP60 are impacted by IBCar treatment. However, RPS27a expression increases by approximately 80% compared to untreated MB468 cells. Knockdown of RPS27a in lung cancer has been reported to inhibit p53 ubiquitination and degradation [83]. RPS27a has also been implicated in mechanisms involving microtubule polymerization inhibitors, including colchicine [84]. Further studies will be needed to explore this aspect of our investigation into the role of RPS27a as part of the multifaceted cytoprotective mechanisms active in normal cells responding to IBCar treatment.

#### 3.7.2. Mitochondrial Membrane Potential

**Figure 14:**
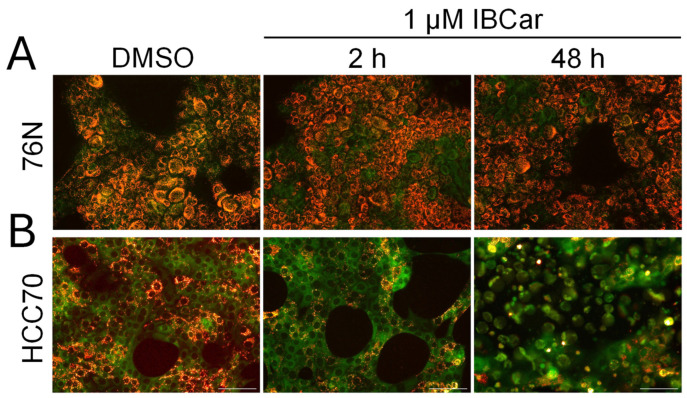
Diverse effects of the IBCar treatment on the mitochondrial membrane potential of normal breast cells 76N (**A**) and BC cells HCC70 (**B**).

Microtubule interactions with mitochondria regulate their intracellular movement, while unpolymerized tubulin modulates ΔΨ in both normal and cancer cells [85,86,87]. Both, depolymerized tubulin as well as discussed above calnexin can contribute to the mitochondrial depolarization. The notion of decreased expression of calnexin in IBCar-treated 76N cells as a contributor to their survival is corroborated by the analysis of changes in ΔΨ (Figure 14A). Mitochondrial ΔΨ was measured using JC-10 dye. In its monomeric form, JC-10 emits green fluorescence; when it accumulates in healthy mitochondria, it forms fluorescent orange aggregates. Mitochondrial depolarization results in the loss of JC-10 accumulation in mitochondria and a shift from orange to green fluorescence indicating compromised mitochondria. Normal 76N cells and BC cells were either treated with 0.1 µM and 1 µM IBCar for 2 h, 24 h, and 48 h or left untreated, DMSO control (Figure 14 and Figure S2). BC cells treated with IBCar rapidly lost mitochondrial membrane potential (ΔΨ), as shown in Figure 14B. This observation aligns with the extensive microtubule depolymerization and the high apoptotic fraction, approaching 100% after 48 h of IBCar treatment, observed in these cells. The increased levels of free tubulin resulting from IBCar-induced microtubule depolymerization lead to significant mitochondrial depolarization in BC cells. By comparison, IBCar has minimal effects on microtubule integrity in normal breast cells, and accordingly, no significant changes in mitochondrial ΔΨ are observed in these cells (Figure 14A). These results were further confirmed using MitoBrilliant^TM^ Live 646, an alternative dye that also depends on mitochondrial ΔΨ (Figure S3).

### 3.8. Efficacy of IBCar in Mice Models of BC

#### 3.8.1. Transgenic Breast Cancer Model

IBCar’s therapeutic potential was evaluated in FVB/N-Tg(MMTV-PyVT)634Mul/J female mice, an established transgenic model of BC. Based on the gene expression profiling, MMTV-PyMT tumors cluster with the luminal B subtype of human breast cancers [88] and recapitulate human BC progression [89,90].

**Figure 15:**
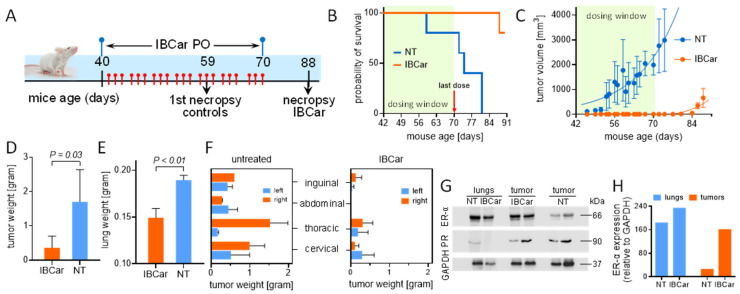
Oral IBCar therapy in MMTV-PyMT female mice. (**A**). Outline of treatment and necropsy schedules. (**B**). Kaplan-Meier survival curves of IBCar-treated and control mice (NT). (**C**). Longitudinal evaluation of tumor volumes. Average weights of tumors (**D**) and lungs (**E**) extirpated from IBCar-treated mice (day 88, i.e., 18 days after the last IBCar dose) and control mice receiving vehicle only (data from mice necropsied on days 59 to 81). (**F**). Comparison of mammary glands involvement in untreated control mice and IBCar-treated mice. Mice treated with IBCar did not develop any mammary gland tumors and lung metastases during treatment. All mammary glands in control mice were involved. (**G**). Expression of ER-α and PR in lysates prepared from tumors extirpated on day 81 (NT mice) and on day 88 (IBCar-treated mice). (**H**). ER-α expression relative of the protein load as measured by GAPDH bands.

IBCar treatment started when mice reached the age of 40 days. Mice were monitored daily, body weight determined, and mammary glands palpated every other day. When the first palpable tumor was detected, all nodules were measured every other day. The formation of pulmonary lesions was determined at necropsy. All findings are summarized in Figure 15. Prolonged treatment with oral IBCar was not associated with any adverse events. Oral IBCar was effective in controlling tumor development (Figure 15B,C). IBCar-treated mice did not develop any mammary gland tumors and lung metastases during treatment. The first signs of mammary tumor development were evident on day 81, i.e., 12 days after the secession of IBCar treatment. In IBCar-treated cohort, we did not detect any tumors in the abdominal mammary gland (Figure 15F).

All control mice developed multiple tumors by 6 weeks of age, which as the disease progressed, involved all mammary glands. In addition, control mice developed numerous lung metastases. Lungs excised from untreated mice exhibited macroscopically visible tumor nodules and were, on average, more than 30% heavier than those from IBCar-treated mice (Figure 15E).

IBCar-treated tumors retained high levels of estrogen receptor-α (ER-α) (Figure 15G,H). The presence of ER-α in BC usually denotes a better prognosis. PyTV tumors, similar to the luminal B subtype of breast cancer, lose ER-α expression as the disease progresses, thereby mimicking the behavior of human breast cancers associated with poor prognosis [89,90]. Loss of ER-α expression frequently results in a more aggressive phenotype and resistance to endocrine therapy [91]. IBCar treatment did not appear to have any major effects on the expression of progesterone receptor.

Standard serum chemistry and hematology panels commonly used to evaluate safety of various compounds in mice were employed to assess potential differences between IBCar-treated and control mice. All measured values were within normal ranges (Table 3 and Table S7). Although glucose levels were somewhat elevated, they remained within the normal range, considering a standard deviation of 47.2 mg/dL. Glucose levels in mice are inherently variable and influenced by factors such as sex, age, strain, and feeding status. In this study, mice had unrestricted access to food and water. Reference data from the Centre for Phenogenomics for 16-week-old female mice report glucose values ranging from 173.0 to 375.5 mg/dL [92]. Overall, hematology and serum chemistry profiles in IBCar-treated mice at study termination were normal, indicating that prolonged IBCar treatment is not toxic to the host.

#### 3.8.2. Subcutaneous Breast Cancer Model MDA-MB-468

**Figure 16:**
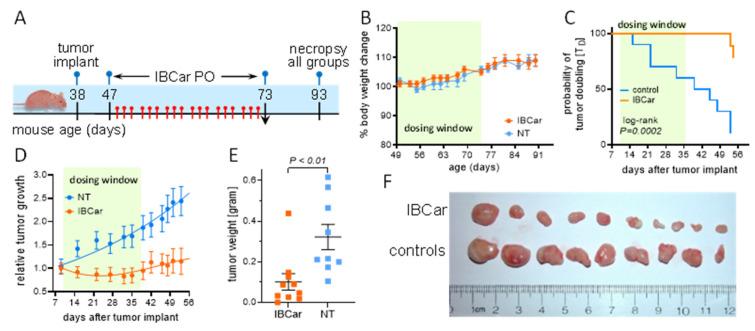
Oral IBCar therapy in immunodeficient J:Nu female mice bearing subcutaneous human MB468 triple-negative breast cancer xenografts. (**A**) Outline of treatment and necropsy schedules. (**B**) Changes in body weights relative to weights one week before xenograft implant. Tumor volumes are subtracted from body weights. NT = control mice. Orally administered IBCar had no adverse impact on the body weight. (**C**) The Kaplan-Meier survival curves of IBCar-treated and control mice. Only two xenografts in mice treated with IBCar doubled their volume during treatment. (**D**) Longitudinal evaluation of tumor volumes. (**E**,**F**) Comparison of tumors extirpated from IBCar-treated mice and vehicle-treated control mice. IBCar-treated tumor weights are >3× smaller compared to tumors from the untreated control mice.

IBCar therapy in TNBC model was conducted in female J:Nu mice. The experimental timeline is shown in Figure 16A. Treatment commenced 10 days after the MB468 cells implants when the average tumor volume (T_V_) was 118 ± 40 mm^3^. Mice were randomized into untreated (NT; T_V_ = 114 ± 15 mm^3^) and IBCar-treated (T_V_ = 118 ± 12 mm^3^) groups. IBCar-treated mice maintained healthy body weights throughout treatment (Figure 16B). Median tumor doubling time determined using the Kaplan-Meier estimator was 42 d in NT mice (Figure 16C). In the IBCar-treated cohort, Tv of two xenografts doubled approximately three weeks after termination of treatment. Analyses of the MB468 tumor growth curves (Figure 16D and Figure S8) indicated a significant delay of tumor growth in IBCar-treated mice with T_D_ > 700 days compared to T_D_ of 33 ± 1.7 days in untreated controls (*p* < 0.0001). Extirpated IBCar-treated tumor weights were more than 3× lower compared to tumors from the untreated mice (Figure 16E,F). Xenograft volumes consistently decreased throughout the IBCar treatment period (days 9 to 35). Tumor regrowth after the secession of IBCar treatment was not significant; T_D_ = 118 ± 12 mm^3^ at the initiation of treatment compared to T_D_ = 135.6 ± 29.3 mm^3^ on day 53 after the BC implant (*p* = 0.8). Furthermore, following the cessation of IBCar treatment (days 35 to 53), no significant tumor regrowth was observed. In contrast, tumor volume in untreated controls increased by more than 240% over the course of the study, whereas the change in tumor volume in IBCar-treated mice was limited to just ~4% and only after treatment had ended. Similarly to PyTV mice, hematology and serum chemistry profiles of IBCar-treated mice at necropsy were normal indicating that prolonged IBCar therapy is not toxic in this mouse strain (Tables S6 and S7).

## 4. Discussion

Breast cancer chemotherapy has long been associated with a range of negative side effects, including chemotherapy-induced peripheral neuropathy (CIPN) and a persistent decline in memory and cognitive function [29,30,31,32,93]. CIPN is one of the most challenging side effects of chemotherapy and a major limiting factor in BC treatment [28]. Nearly 40% of BC patients treated with conventional chemotherapeutics develop CIPN, which not only reduces quality of life but often leads to dose reductions or early cessation of chemotherapy. Both outcomes can adversely impact treatment effectiveness and patient survival. Additionally, nearly 80% of BC patients experience cognitive impairment shortly after starting chemotherapy, which can result from a single treatment or accumulate over the course of multiple drug regimens. Currently, there are approximately 4 million BC survivors in the United States, including women who have completed therapy and those still undergoing treatment [94]. Many of these survivors continue to cope with side effects of their therapies.

Developing drugs with fewer side effects is an important area of research in BC. Data presented in this study strongly support our hypothesis that IBCar is a highly potent, safe, and orally bioavailable agent with efficacy across multiple BC models, both in vitro and in vivo. Notably, one of the most promising features of IBCar is its selective toxicity. It appears to effectively eliminate BC cells while sparing normal cells, which exhibit marked resistance to IBCar.

The heterogeneity of BC, including variability in tumor doubling times and cell cycle parameters, poses a significant challenge to effective treatment. The S-phase fraction of BC tumors can range from 5% to 30% [95,96,97]. Studies measuring BC tumor growth through serial mammograms have reported T_D_s ranging from 44 days to more than 1800 days [98], which is substantially longer than T_D_s of BC cells grown in tissue culture or mouse models. Cell lines selected for this study span a broad range of T_D_s, from approximately 30 h to over 100 h. After short treatment times in vitro, substantial differences in IBCar cytotoxicity among cell lines were observed. However, when the IBCar exposure was extended to 48 h and beyond, these differences became insignificant and did not correlate with T_D_s. It is evident that when therapeutic concentrations are maintained, IBCar demonstrates equivalent efficacy in BC cells with both short and long T_D_s, a finding with important clinical implications. IBCar was also effective in mice with tumor T_D_s ranging from approximately 10 days to 33 days. Furthermore, IBCar’s large therapeutic index, exceeding 50, 100, and 120 relative to pericytes, astrocytes, and normal breast cells, respectively, suggests that achieving effective steady-state concentrations in patients is feasible.

Mutated *TP53* is present in approximately 30% of all breast cancer cases [99] and is one of the most frequently mutated genes in BC. This figure rises to almost 80% in metastatic BC and TNBC [100,101,102]. Given this high prevalence and the well-established role of mutant p53 in promoting drug resistance, *TP53* mutation status was included as an experimental variable. Of the eight cell lines tested in this study, four harbor mutated *TP53*. The entry of all tested BC and normal breast cells into mitosis was found to be regulated by the cdc2 kinase activation irrespective of *TP53* status. Therefore, we propose that the sensitivity of BC cells to IBCar as well as the relative resistance of normal breast cells to IBCar are not linked to the *TP53* status. This is consistent with published reports showing that *TP53*-knockout MCF-10A cells exhibit no significant differences compared to *TP53*-parental MCF-10A cells in response to the clinically used anti-microtubule agent paclitaxel [103]. Mebendazole treatment induces mitotic catastrophe in *TP53*-knockout MB175 cells, while apoptosis is the primary mode of cell death in parental cells [43].

To further investigate the cause of normal cell resistance to IBCar, we examined the differences in death mechanisms between BC and normal breast cells treated with IBCar. All BC cells except MB175 showed evidence of the apoptotic response to IBCar. MB175 cells likely undergo mitotic catastrophe, as evidenced by nuclear fragmentation and micronucleation. While it is well-established that a defective or abrogated G2 checkpoint is crucial for DNA damage-induced mitotic catastrophe, and that the eventual mode of cell death depends on the on the p53 status, the pathways driving this form of cell death in the absence of DNA damage are still largely unknown. MCF-10A cells, which originated from a benign fibrocystic breast disease and spontaneously immortalized, underwent the apoptotic death when exposed to IBCar for 48 h or longer. In contrast, 76N normal breast cells, even after prolonged exposure to IBCar, show no apoptotic population, as confirmed by Cas-3/7 assays and Western blot. The dissimilarities are likely the result of different death pathways.

Untreated normal breast cells express full-length Cas-8, whereas Cas-8 is undetectable in several BC cells. During activation, Cas-8 can cleave itself or nearby Cas-8 molecules at Asp374, leading to the generation of the 41 kDa and 43 kDa fragments. These fragments correspond to the intermediate products in the activation cascade [52,104]. Both of these fragments retain enzymatic activity and contribute to downstream signaling in apoptosis [51,52,53]. During apoptosis, Cas-8 substrates, including the downstream effector caspases Cas-3 and Cas-7, are cleaved and activated by the p41 and p43 Cas-8 intermediates [55]. Fragments p41 and p43 were detectable only in MB361 and MB468 cells, which also exhibit significantly elevated levels of cleaved Cas-3 and activated Cas-3/7. The notable absence of p41 and p43 fragments in 76N cells, despite the prominent presence of full-length Cas-8, confirmed that apoptosis as a death mechanism is not active in these cells. These data suggest dissimilarities in Cas-8 expression and maturation as the potential reasons for disparate responses of normal breast cells and BC cells to IBCar.

Looking deeper into the function of Cas-8 in cellular responses to IBCar, we also analyzed differences in the several components of the normal and BC cells riptosome. RIP1 activities control apoptosis, necroptosis, and inflammatory pathways and are defined by a strict balance between RIP1 activation, fragmentation and degradation, processes that are crucial for maintaining cellular homeostasis [57,58,59,60]. Interactions of RIP1 with Cas-8 are critical to the cell survival and death. High levels of Cas-8 can promote apoptosis by allowing Cas-8 self-cleavage into its active forms. This leads to the activation of downstream pathways. The heterodimerization of Cas-8 can either promote cell survival through RIP1 cleavage or block apoptotic cell death and promote RIP3-mediated necroptosis [105]. Cas-8 plays a significant role in inhibiting necroptosis by cleaving and inactivating RIP1 and RIP3, upstream kinases in the necroptosis pathway. By cleaving RIP1 and RIP3, Cas-8 prevents the phosphorylation and activation of MLKL allowing cells to undergo apoptosis rather than necroptosis. Genotoxic stresses can induce riptosome formation and trigger necroptosis by activation of RIP3-MLKL-dependent necrosis signaling pathways. When Cas-8 is active, it can cleave RIP1 preventing signaling through the necroptotic pathway and promoting apoptosis such as observed in BC cells MB361 and MB468. Conversely, when the Cas-8 activity is inhibited or absent, e.g., in normal breast cells, RIP1 can promote cell survival demonstrating its dual role depending on the cellular context. Even though RIP1 cleavage is primarily mediated by Cas-8, the process can be executed by Cas-3 or Cas-6 in cell lacking Cas-8 [63]. The absence of activated Cas-3 can attenuate RIP1 proteolysis [64], which can also be blocked by mutation of Asp180 [59]. Correspondingly, multiple studies have shown that inhibition of RIP1 cleavage at the kinase domain leads to cell death [59,67,106,107]. The C-terminal cleavage of the RIP1 death domain promotes apoptosis [108,109]. The RIP1 fragmentation at the N-terminus observed in normal breast 76N cells suppresses the apoptotic and necrotic RIP1 activities and promotes cell survival [59]. Vinblastine, which, like IBCar, depolymerizes microtubules, can amplify the expression of necrosome components RIP1, RIP3, and MLKL [66]. IBCar, despite its similar effects on microtubules, does not have any significant impact on the expression of these three proteins in examined BC cells. The RIP1 cleavage site seems to have a decisive role in determining cellular fate. IBCar treatment increases the N-terminal fragmentation of RIP1 in 76N and MB361 cells. Necrostatin-1, a RIP1 inhibitor, rescues RIP1-expressing MB361 BC cells from IBCar-induced cytotoxicity, further confirming RIP1’s role in mediating cell survival or death. Overall, RIP1 appears to play a significant role in mediating the cellular responses to IBCar, influencing its therapeutic efficacy and selective toxicity. Based on the data reported here, we can conclude that the mechanism, which appears to be functional in normal breast cells treated with IBCar, involves pro-survival activities associated with high levels of N-terminal RIP1 kinase fragments, which might activate survival signaling rather than death. We have undertaken a comprehensive evaluation of the full riptosome responses to IBCar in both normal and malignant cells.

Assessment of microtubule integrity provided further insight into the contrasting responses of normal breast and BC cells. IBCar causes permanent microtubule depolymerization in BC cells, whereas in normal breast cells, this effect appears to be transient. The reversibility of IBCar’s effect on microtubules in normal cells contrasts with that of VCR, which induces permanent microtubule damage in both BC and normal cells. Both VCR and IBCar depolymerize microtubules, however, in normal 76N cells treated with IBCar, microtubule integrity is restored within hours after the drug-containing medium is replaced with fresh medium. A few days after IBCar treatment, recovery seems complete, and cells resume normal division rates. Conversely, normal cells treated with VCR sustain permanent damage and die. Anti-microtubule agents can induce apoptosis throughout all phases of the cell cycle, including in non-cycling cells [110,111,112]. Notably, cell death can occur independently of mitosis; for instance, this is a primary contributor to neurotoxicity in patients receiving these agents. While evidence from in vitro and xenograft models supports mitosis as the key phase for the action of microtubule-disrupting drugs [113,114], cancer cells display a broad spectrum of responses to antimitotic therapies [115,116]. In the case of IBCar, the restoration of microtubule integrity in normal cells does not appear to correlate with changes in the mitotic fraction, suggesting that more detailed studies are needed to fully elucidate the mechanisms driving this recovery.

We have identified additional contributors to the differences between BC and normal cells responses to IBCar that involve ER stress response mechanisms and changes in the mitochondrial membrane potential. Mitochondrial depolarization induced by colchicine, which has a similar effect on microtubules as IBCar, is caused by free tubulin released from depolymerized microtubules [87]. This may occur through the insertion of the negatively charged extended C-terminal tail of tubulin into the voltage-dependent anion channel [117], affecting mitochondrial Ca^2+^ exchange, a critical process for balancing cell death, energy demands, and membrane potential. BC cells treated with IBCar rapidly lose mitochondrial ΔΨ consistent with extensive microtubule depolymerization and high apoptotic fraction in these cells. The increased levels of free tubulin produced by IBCar-induced microtubule depolymerization trigger significant depolarization of mitochondria in BC cells. By comparison, IBCar treatment has only a minimal effect on microtubule integrity in normal breast cells with the corresponding marginal changes of ΔΨ.

Disruptions in protein folding induce an unfolded protein response aimed at alleviating the cellular injury. BiP plays a key role in the ER stress responses [118]. Overexpression of BiP inhibits apoptosis in BC cells, reduces chemosensitivity, and appears to contribute to chemoresistance [70]. BiP’s pro-survival functions in the unfolded protein response include targeting misfolded proteins for degradation, binding of Ca^2+^ in ER, and regulating the activation of transmembrane ER stress sensors. When tumors become refractory to therapy, chemotherapeutic agent-induced ER stress activates adaptive unfolded protein response pathways, which work to mitigate the stress and promote chemoresistance [68,69]. Calnexin and BiP interact as sequential chaperones [119,120,121]. This collaboration ensures proper folding and prevents misfolded proteins from leaving the ER. Normal breast 76N cells, both untreated and IBCar-treated, express minimal levels of BiP whereas, untreated BC cells, such as BT549 and MB175, exhibit high intrinsic levels of BiP. Several of tested BC cell lines show a significant increase in BiP synthesis in response to IBCar treatment. These findings indicate that ER stress pathways play a significant role in the response of BC cells to IBCar. Calnexin regulates mitochondrial energy production and stress responsiveness by controlling the activity of Ca^2+^ import into the ER and its transfer to mitochondria. While calnexin levels remain stable in BC cells following IBCar treatment, they decrease in IBCar-treated 76N cells, which appear to maintain mitochondrial membrane potential (ΔΨ) to prevent cell death. This supports the hypothesis that reduced calnexin expression promotes cell survival in 76N cells.

ER stress sensors that mediate the unfolded protein response include PERK, eIF2α, and IRE1α. PERK and IRE1α independently monitor the balance between protein load and folding capacity, regulating key unfolded protein response signal transduction pathways. eIF2α, a downstream effector of PERK, links ER stress signals to the inhibition of translation [77]. Based on our results, IBCar appears to be the cellular ER stressor. Substantial increases in the total PERK expression are detected in 76N and MB361 cells post-IBCar treatment followed by a significant decline at later times. eIF2α is detectable exclusively in 76N normal breast cells suggesting that in normal breast cells the activation of PERK and eIF2α may have cytoprotective functions absent in the BC cells. A recent study identified eIF2α as the pro-survival factor during paclitaxel treatment in vitro and in vivo [79]. Others suggested that apoptosis induced by taxanes is a process downstream of the activation of PERK-eIF2α signaling pathways [80]. IRE1α and p-Ser724-IRE1α are significantly upregulated in 76N cells treated with IBCar, suggesting that two independent unfolded protein response pathways may be active in this cell line. The duration of PERK and IRE1α signaling can vary following disruptions in the protein folding processes. Sustained periods of PERK activities are detrimental to cell survival. The corresponding duration of IRE1α signaling is not [81,82]. Therefore, the transient PERK activity in 76N cells is suggestive of cytoprotective functions whereas the extended PERK activity in MB468 appears to contribute to BC cell death [82]. This temporal regulation of ER stress signaling may be a contributing factor to IBCar’s selectivity. Extensive additional studies will be required to correlate the longitudinal interplay between these two pathways.

IBCar demonstrates strong therapeutic potential across in two subtypes of breast cancer, effectively inhibiting tumor development and progression in both the luminal B and TNBC models. Luminal B tumors exhibit moderate ER expression, elevated levels of proliferation and cell cycle genes, including high Ki67 expression [122]), making them more aggressive than luminal A and resulting in higher incidence of bone metastasis compared to TNBC, 5.64 ± 0.01% for luminal B vs. 2.01 ± 0.01 for TNBC [123]. With current treatments, the 5-year survival rate for patients with metastatic luminal B breast cancer is only 29%, and the median survival is 36 months [124]. Adjuvant systemic chemotherapies for these patients typically include anthracycline-based chemotherapy followed by paclitaxel or docetaxel, anti-microtubule taxane-based regimens [125,126]. In the luminal B subtype, represented by the MMTV-PyMT transgenic mouse model, IBCar completely prevented tumor formation during treatment and significantly delayed recurrence after treatment withdrawal. The median overall survival for metastatic TNBC ranges from 10.2 to 18.7 months, with a 5-year survival rate of around 11% [1,4,5,6,127,128]. Overall prognosis for patients with metastatic TNBC is worse than for the other BC subtypes, and more effective therapeutic options are needed [129]. In the TNBC MB468 xenograft model, oral IBCar administration consistently induced tumor shrinkage and suppressed regrowth post-treatment. These findings suggest that IBCar could serve as a promising alternative or complementary therapy for patients with limited treatment options.

## 5. Conclusions

Most anticancer drugs, including microtubule-targeting chemotherapeutics, discriminate poorly between normal and cancer cells. However, in the instance of IBCar, the contrasting responses are striking. IBCar is selectively more cytotoxic in BC cells. Responses of BC cells are concentration and duration of exposure dependent. To date, we have found no evidence that *TP53* status influences IBCar’s cytotoxicity; the compound is effective in both wt*TP53* and m*TP53* BC cells. In normal cells, protective mechanisms appear to include the reversible nature of IBCar-induced microtubule depolymerization, prosurvival adaptations in the riptosome, and effective management of the ER stress. Our ongoing research focuses on further characterizing the mechanisms underlying these divergent responses. When administered orally, IBCar reduces tumor development and growth in two aggressive BC mouse models with sustained effects even after treatment cessation. IBCar seems to be well tolerated as indicated by the stable body weights and normal hematology and serum chemistry profiles, characteristics that support its potential as a promising and less toxic alternative to conventional anti-microtubule breast cancer therapies.

## Figures and Tables

**Table 1:** Binding energies (E_B_) and inhibition constants (K_i_) estimated in the docking studies.

Compound	E_B_ [kcal/mol]	K_i_ [nM]
colchicine	−9.86	59.0
mebendazole	−7.90	1630.0
**IBCar**	**−11.44**	**4.08**

**Table 2:** Cytotoxicity of IBCar in breast cancer cell lines and normal epithelial breast cells.

		GI_50_ [nM]		GI_50_ [nM]
	96-Well MTS Assay			Clonogenic
Cell Line	24 h	48 h	72 h	48 h Treatment
BT-549	57.5 ± 6.0	14.8 ± 1.2	12.9 ± 1.3	22.9 ± 1.4
HCC70	177.5 ± 16.2	33.7 ± 6.9	17.8 ± 2.2	12.3 ± 2.8
MDA-MB-175-VII	nd *	172.4 ± 36.5	24.2 ± 3.5	24.0 ± 6.6
MDA-MB-231	nd	41.4 ± 7.8	nd	19.5 ± 3.4
MDA-MB-361	nd	21.9 ± 3.1	nd	3.0 ± 2.1
MDA-MB-468	nd	25.3 ± 1.5	nd	7.3 ± 1.9
MCF-10A	1149 ± 357	149.9 ± 49.7	nd	nd
76N	2561 ± 315	1786 ± 389	1622 ± 580	nd

* nd = not determined.

**Table 3:** FVB/N-Tg(MMTV-PyVT)634Mul/J female mice serum chemistry in IBCar-treated and controls mice.

		IBCar	Control	Standard Values
Parameter	Units	Average (*std dev*)	Average (*std dev*)	Range
Albumin (ALB)	g/dL	4.4 (*0.3*)	4.7 (*0.4*)	2.5–4.9
Alkaline phosphatase (ALP)	U/L	90.0 (*11.1*)	68.0 (*19.9*)	16–291
Alanine aminotransferase (ALT)	U/L	84.3 (*43.0*)	57.0 (*17.6*)	7–277
Amylase (AMY)	U/L	847.8 (*92.3*)	876.7 (*73.4*)	na *
Bilirubin, total (total bil)	mg/dL	0.4 (*0.1*)	0.3 (*0.1*)	0.1–1.1
Bood urea nitrogen (BUN)	mg/dL	17.8 (*1.3*)	18.0 (*2.6*)	2–71
Calcium (Ca)	mg/dL	11.3 (*0.9*)	11.9 (*0.2*)	6.8–11.9
Phosphorus (PHOS)	mg/dL	11.8 (*0.6*)	11.0 (*1.3*)	5.3–11.3
Creatinine (CRE)	mg/dL	0.4 (*0.1*)	0.3 (*0.1*)	0.1–2.1
Glucose (GLU)	mg/dL	310.3 (*47.2*)	258.7 (*12.1*)	173–376
Sodium (Na^+^)	mmol/L	157.3 (*11.1*)	160.0 (*4.4*)	145–176
Potassium (K^+^)	mmol/L	8.5 (*0.0*)	8.5 (*0.0*)	6.5–9.7
Total protein (TP)	g/dL	5.4 (*0.5*)	5.9 (*0.5*)	3.3–7.6
Globulin (GLOB)	g/dL	1.1 (*0.2*)	1.2 (*0.2*)	na

* not available.

## Data Availability

Supplementary Materials attached to this article at https://www.mdpi.com/article/10.3390/cancers17152526/s1 provide access to data not included in the article. Other data presented in this study are available on request from the corresponding author due to some restrictions related to intellectual property.

## Supplementary Materials

The following supporting information can be downloaded at: https://www.mdpi.com/article/10.3390/cancers17152526/s1, Details of several experimental protocols. The uncropped bolts. Figure S1: Molecular docking of IBCar regioisomers. Figure S2: Analyzed Western blot protein band intensities. Figure S3: IBCar-concentration-dependent evaluation of mitochondrial potential in 76N normal breast cells using MitoVolt and time-dependent evaluation of mitochondrial potential in 76N normal breast cells treated with 1 µM IBCar as compared to the DMSO controls using Mito Brilliant. Figure S4: Western immunoblot of cleaved PARP in BC cells untreated (NT) and IBCar-treated (500 nM IBCar, 24 h). Figure S5: Western immunoblot of Western immunoblots of histone (Ser-10). Figure S6: Confirmatory Western immunoblot of Cas-8 in lysates from BC cells untreated (NT) and IBCar-treated (500 nM IBCar, 48 h) using rabbit anti-human, mouse, rat-Cas-8 antibodies PA5-87373. Figure S7: Human HSP arrays results (shown in Figure 1) of lysates from BC cells. Figure S8: Longitudinal changes in tumor volume in control mice and IBCar-treated mice. Figure S9: Nuclear fragmentation in normal and BC cells treated with IBCar. Blue arrowheads indicate fragmented nuclei and micronucleation. Figure S10: Determination of the basal levels of activated Cas-3/7 in normal breast and BC cells. Figure S11: Activated Cas-3/7 populations in HCC70 and MB468 cells treated with 100 nM and 500 nM IBCar. Figure S12: Microtubules in untreated HCC70 breast cancer cells. Figure S13: Remnants of microtubules in HCC70 breast cancer cells treated with IBCar. Figure S14: Microtubules in untreated 76N normal breast cells. Figure S15: Microtubules in 76N normal breast cell treated with IBCar. Table S1: Antibodies used in this study. Table S2: Reagents and supplies for gel electrophoresis and Western blotting. Table S3: Characteristics of human cell lines used in this study. Autodock 4.2 Table of RMSD table for IBCar shown in the manuscript Figure 1. Table S4: Statistical analyses for GI_50_s. Table S5: Statistical analyses of MDA-MB-468 xenograft growth curves. Table S6: Hematology of female mice bearing human breast cancer xenografts. Table S7: Serum chemistry of female mice bearing human breast cancer xenografts. Table S8: GI50 concentrations of mebendazole, vincristine, and IBCar determined by the NCI DTP in several breast cancer cell lines. Table S9: Tumor growth curve analyses in MMTV-PyMT mice. Table S10: Statistical comparison of tumor weights extirpated from MMTV-PyMT mice at the age of 81 days. Table S11: Statistical comparison of survival curves in MMTV-PyMT transgenic mice.
