# Supplementary material for: IBCar: Potent Orally Bioavailable Methyl N-[5-(3′-Iodobenzoyl)-1H-Benzimidazol-2-yl]Carbamate for Breast Cancer Therapy
Source: Cancers (Basel). 2025 Jul 30;17(15):2526. doi: 10.3390/cancers17152526 (PMC12346495; doi:10.3390/cancers17152526)
Supplement: Supplementary file 1 [file cancers-17-02526-s001.zip › cancers-3712430-Supplementary.pdf]

Supplementary Materials to

IBCar: Potent, Orally Bioavailable Methyl *N*-[5-(3′-Iodobenzoyl)-1*H*-Benzimidazol-2-yl]Carbamate for Breast Cancer Therapy

Janina Baranowska-Kortylewicz [1,*] and Ying Yan [2]

[1] Department of Pharmaceutical Sciences, College of Pharmacy, University of Nebraska Medical Center, Omaha, NE 68198-6120, USA
[2] Department of Radiation Oncology, College of Medicine, University of Nebraska Medical Center, Omaha, NE 68198-6861, USA; yyan@unmc.edu
* Correspondence: jbaranow@unmc.edu

Table S1. Antibodies used in this study.

catalog number	antibody	reactivity	host	MW (kDa)	vendor
3177	BiP (C50B12)	H, M	rabbit	78	Cell Signaling Technologies
MBS2539112	BIP (HSPA5) Polyclonal	H	rabbit	75	MyBioSource
2679	Calnexin (C5C9)	H, Mk	rabbit	90	Cell Signaling Technologies
MBS2536336	Calnexin (CANX), Polyclonal	H, M, R	rabbit	90	MyBioSource
14220	Caspase-3 (D3R6Y)	H, M, R, Mk	rabbit	35, 19, 17	Cell Signaling Technologies
9746	Caspase-8 (1C12)	H	mouse	18, 43, 57	Cell Signaling Technologies
9746	Caspase-8 (PA5-87373)	H, M, R	rabbit	18, 43, 57	Thermo Fisher Scientific
8064	CDT1 (D10F11)	H, Mk	rabbit	65	Cell Signaling Technologies
2895	CHOP (L63F7) Mouse mAb #2895	H, M, R	mouse	27	Cell Signaling Technologies
9664	Cleaved Caspase-3 (Asp175) (5A1E)	H, M, R, Mk	rabbit	17, 19	Cell Signaling Technologies
98134	Cleaved Caspase-8 (Asp374) (E6H8S)	H	rabbit	18, 43, 57	Cell Signaling Technologies
9496	Cleaved Caspase-8 (Asp374) (18C8)	H	rabbit	18, 41, 43	
91500	Cyclin A2 (E1D9T)	H	rabbit	55	Cell Signaling Technologies
12231	Cyclin B1 (D5C10) XP®	H, R	rabbit	55	Cell Signaling Technologies
20808	Cyclin E1 (D7T3U)	H, M, R	rabbit	48	Cell Signaling Technologies
5324	eIF2α (D7D3)	H, M, R, Mk	rabbit	38	Cell Signaling Technologies
3398	Phospho-eIF2α (Ser51)	H, M, R, Mk, Dm	rabbit	38	Cell Signaling Technologies
MAB57151	ER-α (NR3A1 antibody)	H, M, R	mouse	65-70	Bio-Techne
13258	ER-α (D6R2W)	H	rabbit	66 kDa	Cell Signaling Technologies
3264	Ero1-Lα Antibody 3264	H	rabbit	60	Cell Signaling Technologies
52508	Geminin (E5Q9S) XP®	H, Mk	rabbit	25	Cell Signaling Technologies
4499	Histone H3 (D1H2) XP® Rabbit mAb #4499	H, M, R, Mk	rabbit	17	Cell Signaling Technologies
21738-1-AP	Integrin-beta 4, Polyclonal	H, M, R	rabbit	200 - 240	Proteintech
3294	IRE1α (14C10)	H, M, R	rabbit	130	ER stress
NB100-2324	IRE1α	H, M, R	rabbit	110	Novus
26539	MLKL (E7V4W)	H, M	mouse	54	Cell Signaling Technologies
2527	p53 (7F5) Rabbit mAb #2527	H, Mk	rabbit	53	Cell Signaling Technologies
3501	PDI (C81H6)	H, M, R, Mk	rabbit		Cell Signaling Technologies
5683	PERK (D11A8)	H	rabbit	140	Cell Signaling Technologies
bsm-51385M	PERK (EIF2AK3) (3C3)	H, M, R	mouse	125	Bioss Antibodies
4539	Phospho-cdc2 (Tyr15) (10A11)	H, M, R, Mk	rabbit	34	Cell Signaling Technologies
3377	Phospho-Histone H3 (Ser10) (D2C8) XP®	H, M, R, Mk, Z	rabbit	17	Cell Signaling Technologies
NB100-2323	Phospho-IRE1 Alpha (Ser-724)	H, M, R, Rb, Mk	rabbit		Novus
91689	Phospho-MLKL (Ser358) (D6H3V)	H	rabbit	54	Cell Signaling Technologies
9284	Phospho-p53 (Ser15) Antibody #9284	H, M, R, Mk	rabbit	53	Cell Signaling Technologies
2521	Phospho-p53 (Ser46) Antibody #2521	H, Mk	rabbit	53	Cell Signaling Technologies
3179	Phospho-PERK (Thr980) (16F8)	R	rabbit	170	Cell Signaling Technologies
93654	Phospho-RIP3 (Ser227) (D6W2T)	H	rabbit	46-62	Cell Signaling Technologies
orb315652	Progesterone Receptor	H, M, R	rabbit	90 (A), 118 (B)	Biorbyt
8757	Progesterone Receptor A/B (D8Q2J)	H	rabbit	90 (A), 118 (B)	Cell Signaling Technologies
3493	RIP (D94C12) XP® Rabbit mAb #3493	H, M	rabbit	78	Cell Signaling Technologies
13526	RIP3 (E1Z1D) Rabbit mAb #13526	H	rabbit	46-62	Cell Signaling Technologies
RGAM001	HRP-Goat anti-Mouse	M	goat		Proteintech
RGAR001	HRP-Goat anti-Rabbit	Rb	goat		Proteintech
7074	HRP anti-Rabbit	Rb	goat		Cell Signaling Technologies
7076	HRP anti-Mouse	M	horse		Cell Signaling Technologies
649202	anti-GAPDH	H	mouse	37	BioLegend
MAB5718	anti-GAPDH	H, M, R	mouse	37	Bio-Techne
8884	HRP-GAPDH conjugate	H, M, R, Mk	rabbit	37	Cell Signaling Technologies

H-Human; M-Mouse; R-Rat; Mk-Monkey; Dm-*D. melanogaster*; Rb-Rabbit

Table S2. **Reagents and supplies for gel electrophoresis and Western blotting**.

catalog number	reagent	vendor
1610375	Precision Plus Protein™ Kaleidoscope™ molecular weight standards	Bio-Rad Laboratories
26619	PageRuler™ Plus Prestained protein ladder, 10 to 250 kDa	Thermo Fisher Scientific
926-98000	WesternSure® Pre-stained Chemiluminescent protein ladder	LI-COR Biotechnology
1610732	10x Tris/Glycine/SDS running buffer	Bio-Rad Laboratories
1703932EDU	Mini Trans-Blot® filter paper	Bio-Rad Laboratories
4561095	4–20% Mini-PROTEAN® TGX™ precast protein gels (12-well, 20 μL)	Bio-Rad Laboratories
4569035	Any kD™ Mini-PROTEAN® TGX™ precast protein gels (12-well, 20 μL)	Bio-Rad Laboratories
4561044DC	12% Mini-PROTEAN® TGX™ precast protein gels (10-well, 50 μL)	Bio-Rad Laboratories
4561034	10% Mini-PROTEAN® TGX™ precast protein gels (10-well, 50 μL)	Bio-Rad Laboratories
IPVH00010	MilliporeSigma™ Immobilon™-P PVDF membrane	Thermo Fisher Scientific
WP20005	Invitrogen™ Novex™ ECL Chemiluminescent Substrate Reagent Kit	Thermo Fisher Scientific
B7703S	Blue Loading buffer pack	New England BioLabs
161-0737	2×Laemmli sample buffer	Bio-Rad Laboratories

Experimental details

Chemicals, Reagents, and Antibodies

Positive control cell lysate standards were purchased from Santa Cruz Biotechnology (Dallas, TX). NucBlue™ Live ReadyProbes™ reagent (Hoechst 33342); LIVE/DEAD™ Cell imaging kit (488/570); Tubulin Tracker™ Deep Red; and CellEvent™ Caspase-3/7 Green ReadyProbes™ reagents were purchased from Thermo Fisher Scientific (Waltham, MA). ION Vital-MitoVolt assay kit was from ION Biosciences (San Marcos, TX). MitoBrilliant™ Live 646 were from BioTechne-Tacris (Minneapolis, MN). Apoptotic, Necrotic and Healthy Cells quantitation kit was purchased from Biotium, Inc. (Fremont, CA). Cells were imaged in Fluorobrite DMEM (Life Technologies Corporation, Carlsbad, CA) or Invitrogen™ Live Cell Imaging solution (Thermo Fisher Scientific). Human HSP Array C1 was from RayBiotech (Norcross, GA). Cell lysis buffer (10×) and protease/phosphatase inhibitors (100×) were from Cell Signaling Technology (Danvers, MA). Pierce micro-BCA protein assay for total protein determination and Halt protease and phosphatase single-use inhibitor cocktails were purchased from Life Technologies Corporation (Carlsbad, CA).

Cell Proliferation, GI_{50} Determination, and Cytotoxicity Assays

Cell Doubling Times. T_Ds were determined using a routine trypan blue exclusion method to count cells at 24 h, 48 h, 72 h and 96 h after plating (Nexcelom Cellometer Auto 1000 Brightfield Cell Counter). Doubling times T_Ds were calculated as: *T_D (hours) = (hours in culture - 24 h) × ln(2)/ln(number of cells harvested at time t/number of cells harvested at 24 h)*.

Cellular Metabolic Activity, MTS Assay and GI_{50} Determination. Cells suspensions at 20,000-50,000 cells/mL (depending on the cell line) were seeded into 96-well plates (technical replicates n=6 per concentration) and allowed to attach for 24 h. A freshly prepared stock solution of IBCar in DMSO was used to prepare IBCar dilutions in full growth medium. Control cells received medium containing DMSO. Cell metabolic activity was analyzed after 24 h, 48 h and 72 h treatment. Spent medium was removed, monolayer washed with PBS, and fresh medium containing the MTS reagent was added. Cells were incubated for 30 min to 120 min under the standard culture conditions. Plates were shaken briefly and absorbance measured at 490 nm using the Opsys MR microplate reader (Dynex Technologies, Chantilly, VA).

Counting Cell Nuclei. Cells grown in medium containing either IBCar or DMSO were washed once with PBS and twice with HBSS containing calcium and magnesium to remove non-adherent and dead cells. Hoechst 33342, a cell-permeant nuclear counterstain that emits blue fluorescence when bound to DNA (NucBlue™ Live ReadyProbes™ Reagent) was used for imaging. Two drops of NucBlue per 1 mL medium were added and cells were incubated for 30 min. At least three fields per concentration were imaged (λ_{ex} = 350 nm; λ_{em} = 460 nm) and nuclei

counted. This method alongside life/dead cell assay described below was used to validate the MTS assay results for the adherent cells that do not form colonies.

Life/Dead Cell Assays. Cells treated with IBCar or DMSO (controls) were washed twice with FluoroBrite™ DMEM. SYTOX™ Green nucleic acid stain, impermeant to live cells, was used as the indicator of dead cells within a cell population. The SYTOX Green staining solution (167 nM) in FluoroBrite™ DMEM and added to cells. Cells were incubated for 30 min protected from light, the staining solution decanted, cells washed twice with FluoroBrite™ DMEM, and imaged (λ_{ex} = 504 nm; λ_{em} = 523 nm) using the FLoid™ Cell Imaging Station (Thermo Fisher Scientific). For the life/dead cell assay and to validate the results of other survival assays, the LIVE/DEAD™ Cell Imaging Kit 488/570 was used in two ways: cells were either imaged with the FLoid™ Cell Imaging Station or the imaging protocol customized for the 96-well microplate fluorescence reader was used (PerkinElmer LS-55 Fluorescent spectrometer with the 96-well plate-reader accessory); λ_{ex} = 488, 570 nm; λ_{em} = 515, 602 nm).

Clonogenic Assay. Cells harvested with trypsin-EDTA (0.25%) were plated into T25 flasks (200-800 cells/flask; n=2 replicates per treatment) and allowed to recover for 48 h. Spent medium was removed and cells were exposed to several concentrations of IBCar for 24 h and 48 h. Drug-containing medium was removed, monolayers washed once with full medium, and fresh growth medium was added. Cells were grown for 14 days to 21 days with fresh medium changes every 7 days. Colonies were stained with crystal violet and either manually counted by two independent observers or analyzed using ImageJ as described previously [40].

Protein Extraction and Western Blotting

Cells grown to 70%-80% confluence were incubated with IBCar (0.5 μM, 1 μM) for 24 h and 48 h. Spent medium was collected, cell monolayers washed twice with PBS. Cell lysates were prepared using commercial lysis buffers supplemented with protease and phosphatase inhibitors. Protein concentration in cell lysates was measured using Micro BCA™ protein assay kit (ThermoFisher Scientific). For gel electrophoresis, cell lysate aliquots were added to the 2× sample buffer. Protein samples were denatured at 95°C for 5 min, cooled, and loaded onto gels at 50-100 μg total protein per well. Gels were run at the constant voltage (150 V) for ~1 h. Proteins were transferred onto a Hybond-P 0.45 μm PVDF membrane (Amersham Biosciences, Piscataway NJ) using a constant current of 30 mA for 18 h at 4°C or 80 mA for 2 h at rt. PageRuler Plus prestained protein ladder, 10 to 250 kDa (Life Technologies Corp) or Precision Plus Protein™ Kaleidoscope™ prestained protein standards (Bio-Rad Laboratories, Hercules, CA) were used as the molecular weight markers and to monitor the efficiency of protein transfer. Membranes were incubated for 1 h at rt or 18 h at 4°C in the blocking buffer containing either 5% w/v nonfat dry milk or 5% bovine serum albumin (fraction V). Blocked membranes were incubated in the blocking buffer containing primary antibodies overnight at 4°C, washed and incubated with HRP-conjugated anti-species secondary antibodies at rt for 2-3 h. The protein load was measured with anti-GAPDH antibodies. Antigens were detected using Novex ECL Chemiluminescent substrate reagent kit according to the manufacturer's instructions (Life Technologies Corp.).

Cell Cycle Analyses

Changes in the cell cycle phases were evaluated in BCa cells and cells derived from normal breast tissue after treatment with various concentrations of IBCar for 24 h and 48 h. Untreated control cells were grown in medium containing DMSO. Cells were harvested using non-enzymatic dissociation buffer; washed twice with PBS and centrifuged at 1,500 rpm for 10 min at 4°C. Cell pellets were resuspended in ice-cold 70% ethanol and gently vortexed to obtain monodispersed cell suspensions. Cell suspensions were stored at -20°C until all samples were ready for the flow cytometry analyses. Ethanol-fixed cells were centrifuged, ethanol decanted, and cells washed one time with PBS. The resultant cell pellets were resuspended, ~1×10^6 - 5×10^6 cells/mL, in the Telford reagent (16.81 mg EDTA diso-dium salt; 13.4 g RNAse A (93 U/mg); 25 mg propidium iodide; 500 μL Triton X-100 in 500 mL PBS). Cell suspensions were kept in the dark at room temperature for 2 h. Stained cell suspensions were transferred to the flow cytometer and cell-associated fluorescence was measured.

Table S3. Characteristics of human cell lines used in this study.

**cell line** (abbreviation)	**disease**	**source**	**race**	***TP53* status**	**T_D [hours]**
**BT-549** (BT549)	TNBC*	primary	White	R249S	57.9±9.6
**HCC70** (HCC70)	TNBC	primary	Black	R248Q	60.0±8.9
**MDA-MB-175-VII** (MB175)	carcinoma	pleural effusion	Black	WT	100.8±8.9
**MDA-MB-231** (MB231)	TNBC	pleural effusion	White	R280K	32.6±6.3
**MDA-MB-361** (MB361)	carcinoma	brain	White	WT	94.1±3.1
**MDA-MB-468** (MB468)	TNBC	pleural effusion	Black	R273H	42.5±3.9
**MCF-10A** (MCF10A)	fibrocystic breast	fibrocystic breast	White	WT	21.0±5.6
**76N** (76N)	epithelial cells	mammo-plasty	not defined	WT	23.0±2.3

* TNBC = triple negative breast cancer

Figure S1. **Molecular docking of IBCar regioisomers**.

This analysis was conducted by Dr. N. Y. Palermo.[1] The structure of tubulin was obtained from the Protein Data Bank (rcsb.org), PDB accession id: 4O2B. Only the α and β chains and colchicine were retained for modeling. The protein was prepared using the Schrodinger Protein Preparation wizard[2] with default settings. Three regioisomers of IBCar were docked into the colchicine binding site using the Induced Fit Docking regime with Glide accuracy set to XP mode. Poses were ranked by the Glide XP score. Meta-IBCar has the highest calculated energy of binding (E_B = -8.829), followed by ortho-IBCar (E_B = -8.752) and para-IBCar (E_B = -8.651). All three regioisomers make hydrogen bonds with α-chain residues Asn 101 and Ser 178 and β-chain residue Lys 254. Ortho- and para-IBCars make halogen bonds with the β-chain, whereas meta-IBCar does not.

Meta-IBCar has the lowest energy of binding despite making no halogen bonds. The iodine in the meta position allows the aromatic ring to fit more deeply in the colchicine site, which outweighs any energetic advantage provided by a halogen bond.

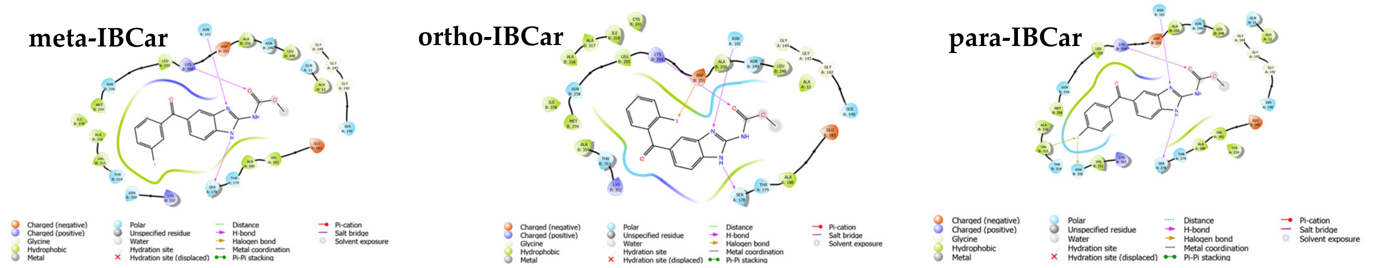

Autodock 4.2 Table of RMSD table for IBCar shown in the manuscript Figure 1.

rank	binding energy	cluster RMSD	reference RMSD
**1**	**-11.44**	**0**	**15.72**
2	-10.69	0	20.20
2	-10.69	0.12	20.16
2	-10.65	0.11	20.21
3	-10.44	0	24.39
3	-10.02	0.95	24.25
4	-10.37	0	21.30
5	-10.19	0	21.38
6	-9.97	0	23.45
7	-9.96	0	20.30

[1] Computational Chemistry Core, University of Nebraska Medical Center, Omaha, NE, USA

[2] Schrödinger Release 2022-1: Schrödinger, LLC, New York, NY, 2021.

Figure S2. Western blot band intensities.

p-cdc2 and GAPDH.

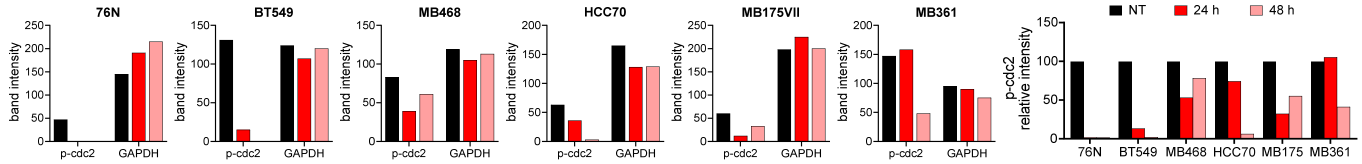

Cleaved Cas-3 and GAPDH.

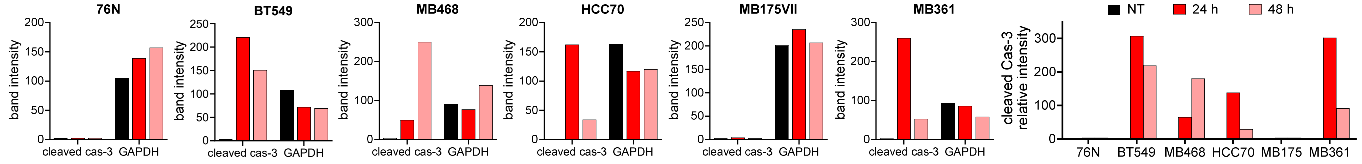

Cas-3 and GAPDH.

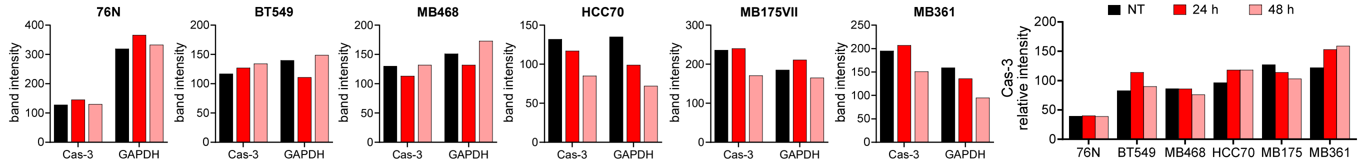

Cas-8 and GAPDH.

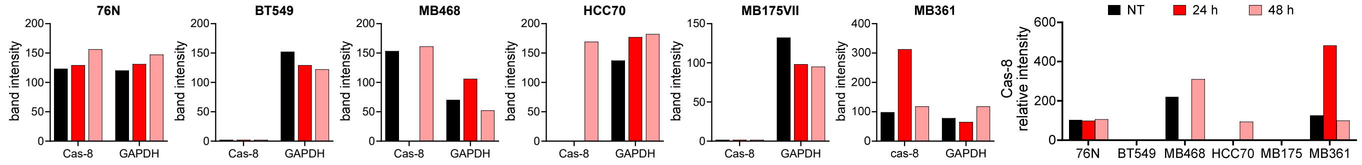

Cleaved Cas-8 and GAPDH.

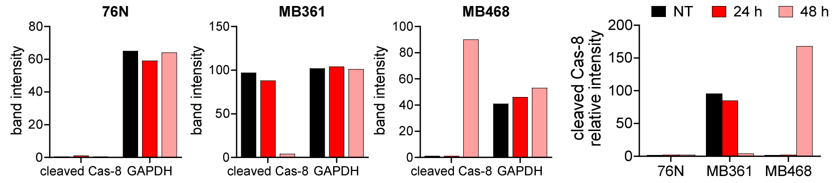

MLKL and GAPDH.

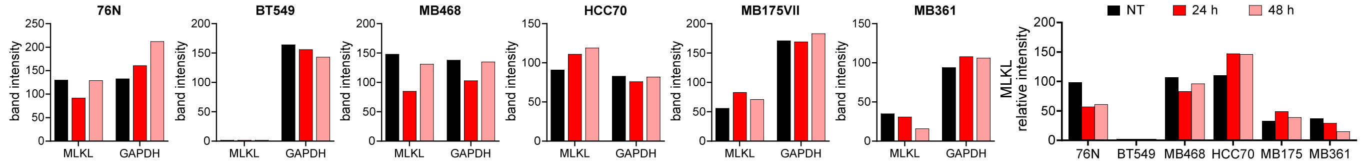

RIP3, p-RIP3 and GAPDH.

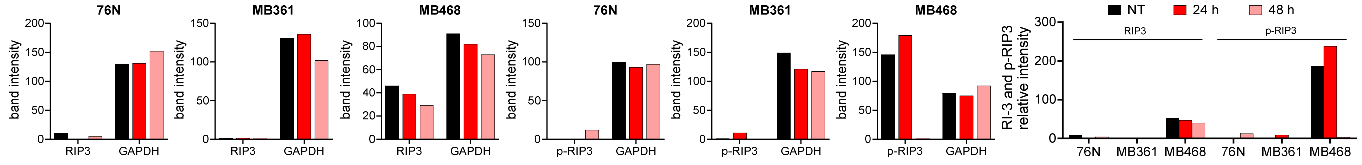

RIP1, p-RIP1 and GAPDH.

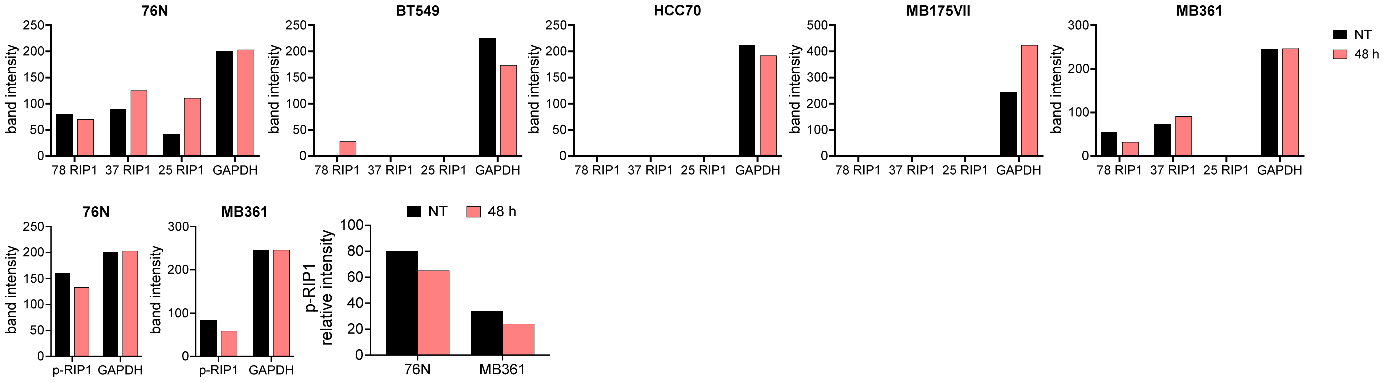

BiP and GAPDH.

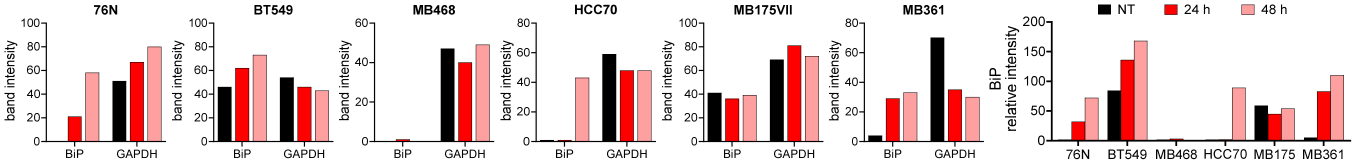

Calnexin and GAPDH.

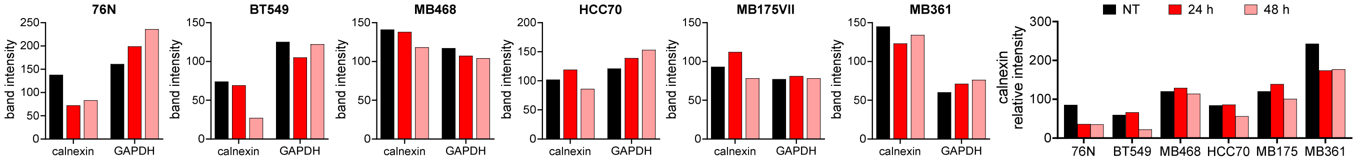

PERK and GAPDH

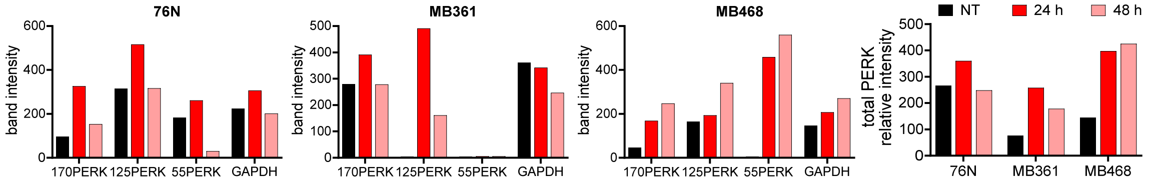

eIF2α and GAPDH

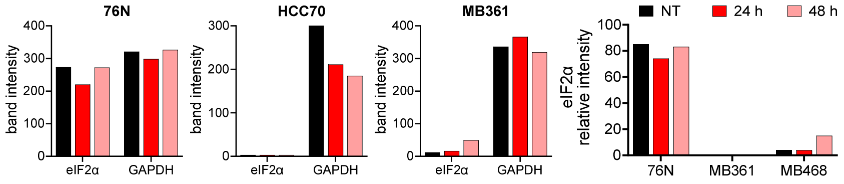

IRE1α and GAPDH

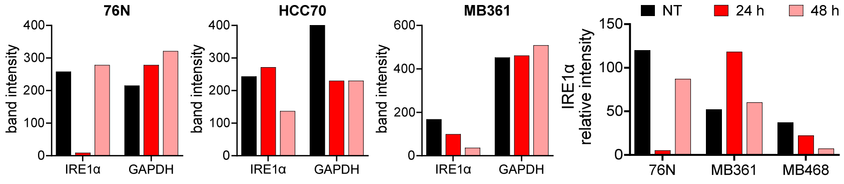

ER-α, PR and GAPDH

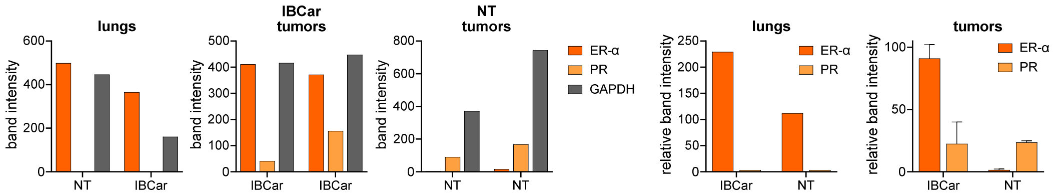
lungs
IBCar tumors
NT tumors
band intensity
relative band intensity
NT
IBCar
ER-α
PR
GAPDH
lungs
tumors
600
400
200
0
500
300
100
800
250
150
50

Table S4. Statistical analyses for $GI_{50}s$.

Descriptive statistics

	BT549	HCC70	MB175	MB231	MB361	MB468	MCF10A	76N
Mean	14.8	33.7	172.4	41.4	21.9	25.9	149.9	1786
Std. Deviation	3.6	20.7	109.5	23.4	9.3	5.7	149.1	1167
Std. Error of Mean	1.2	6.9	36.5	7.8	3.1	1.9	49.7	389
Lower 95% CI	12.03	17.79	88.23	23.41	14.75	21.52	35.29	889
Upper 95% CI	17.57	49.61	256.6	59.39	29.05	30.28	264.5	2683

ANOVA summary

F 19.26
P value <0.0001
Significant diff. among means (P < 0.05)? Yes
R squared 0.8081

Bartlett's test

Bartlett's statistic (corrected) 146.9
P value <0.0001
Are SDs significantly different (P < 0.05)? Yes

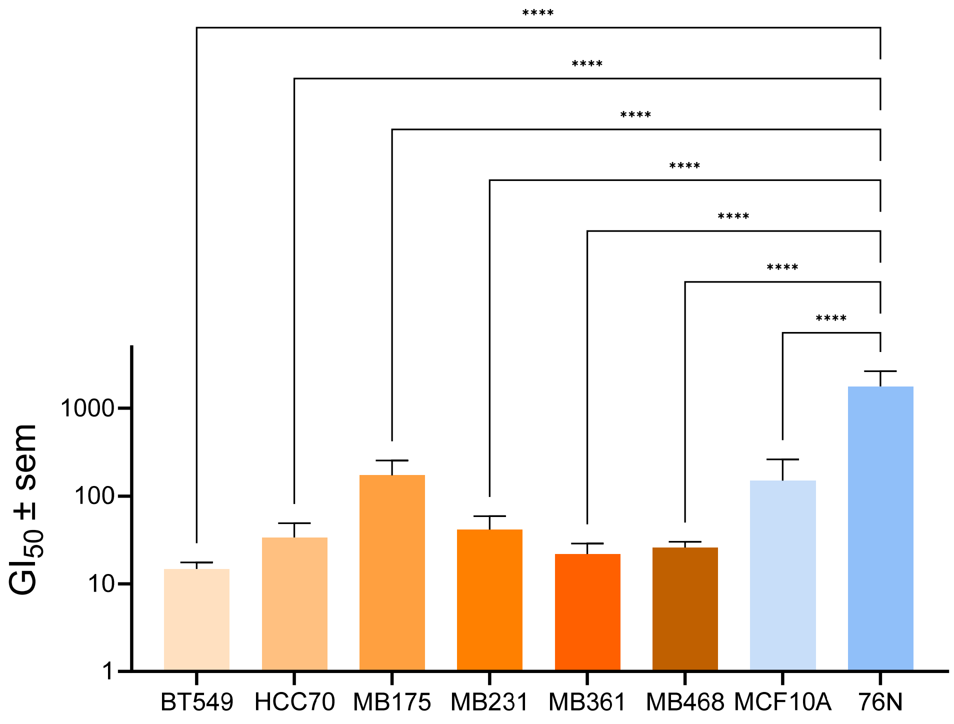

Figure S3. **IBCar-concentration-dependent evaluation of mitochondrial potential** in 76N normal breast cells using MitoVolt and time-dependent evaluation of mitochondrial potential in 76N normal breast cells treated with 1 μM IBCar as compared to the DMSO controls using Mito Brilliant.

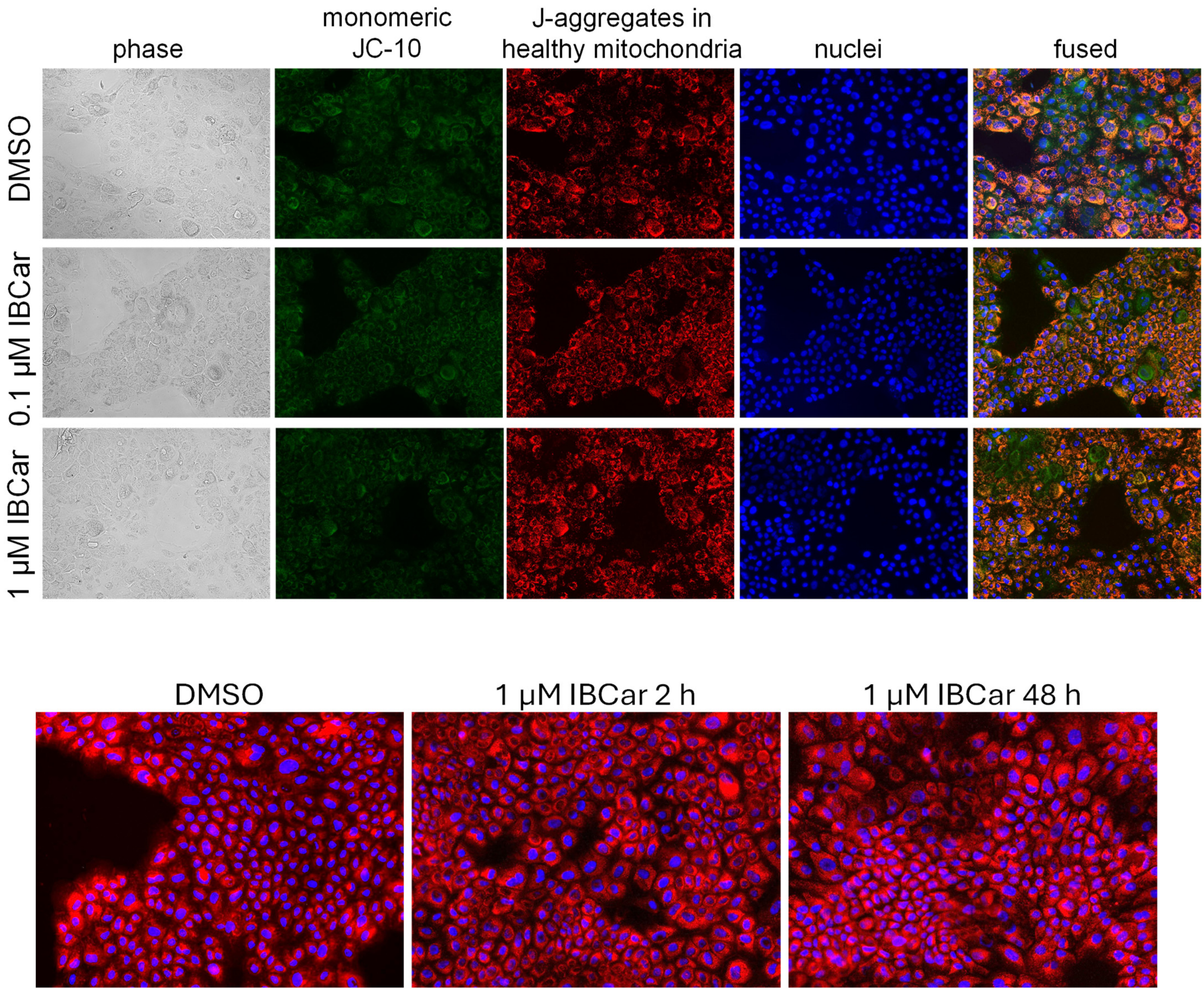

Figure S4. **Western immunoblot of cleaved PARP** in BCa cells untreated (NT) and IBCar-treated (500 nM IBCar, 24 h).

Sample: BT-549 NT, BT-549 500, HCC70 NT, HCC70 500, MB-175 NT, MB-175 500, MB-361 NT, MB-361 500
Antibody: cleaved PARP(D214)
Calculated MW (kDa): 113

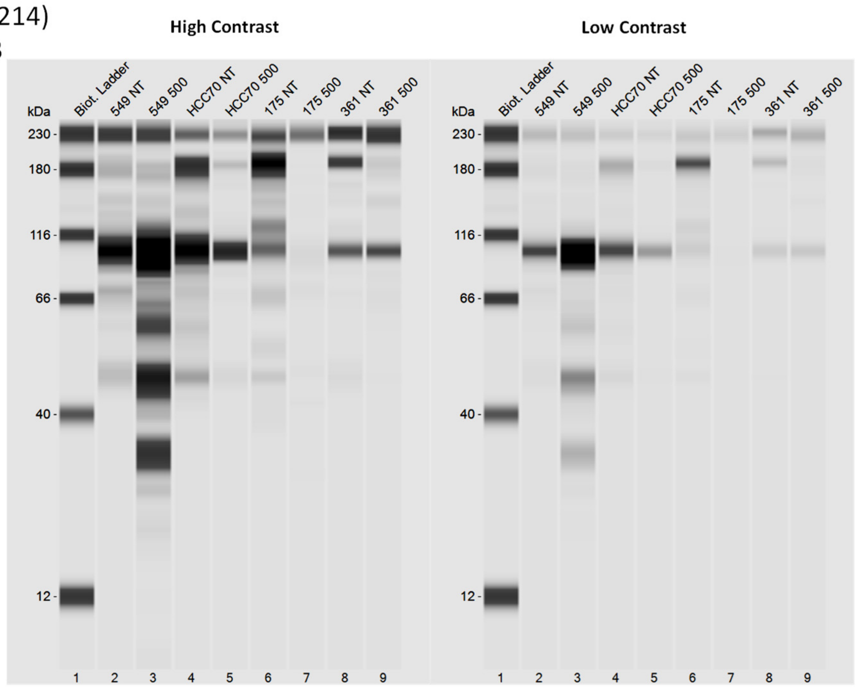

cleaved PARP(D214) Positive Control

Sample: Hela+STS
Antibody: cleaved PARP(D214)
Calculated MW (kDa): 113

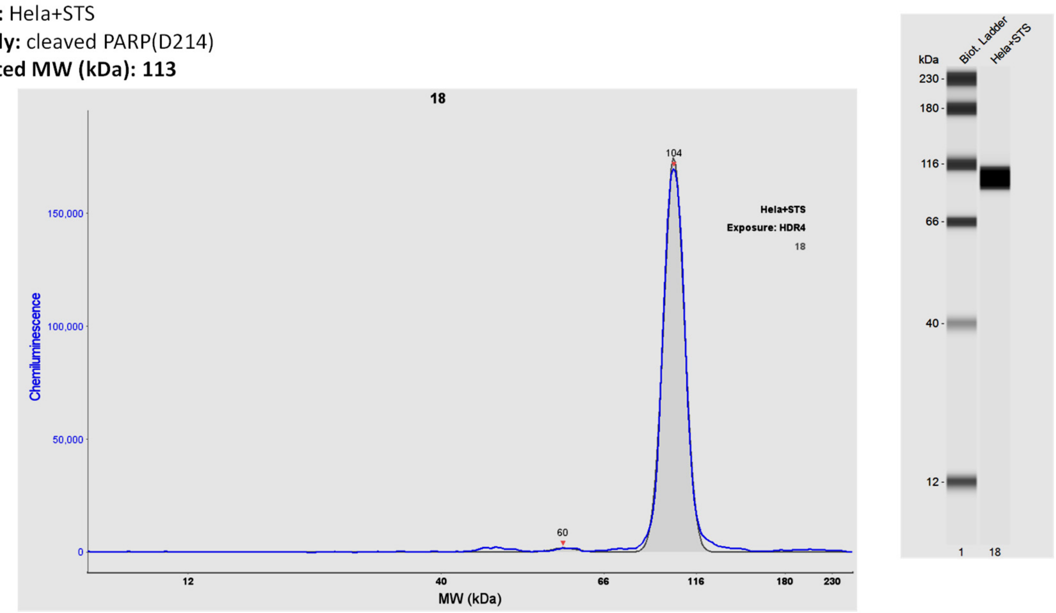

Figure S5. **Western immunoblot of histone (S10)**. The right panel shows positive control data: HeLa cells treated with Nocodazole.

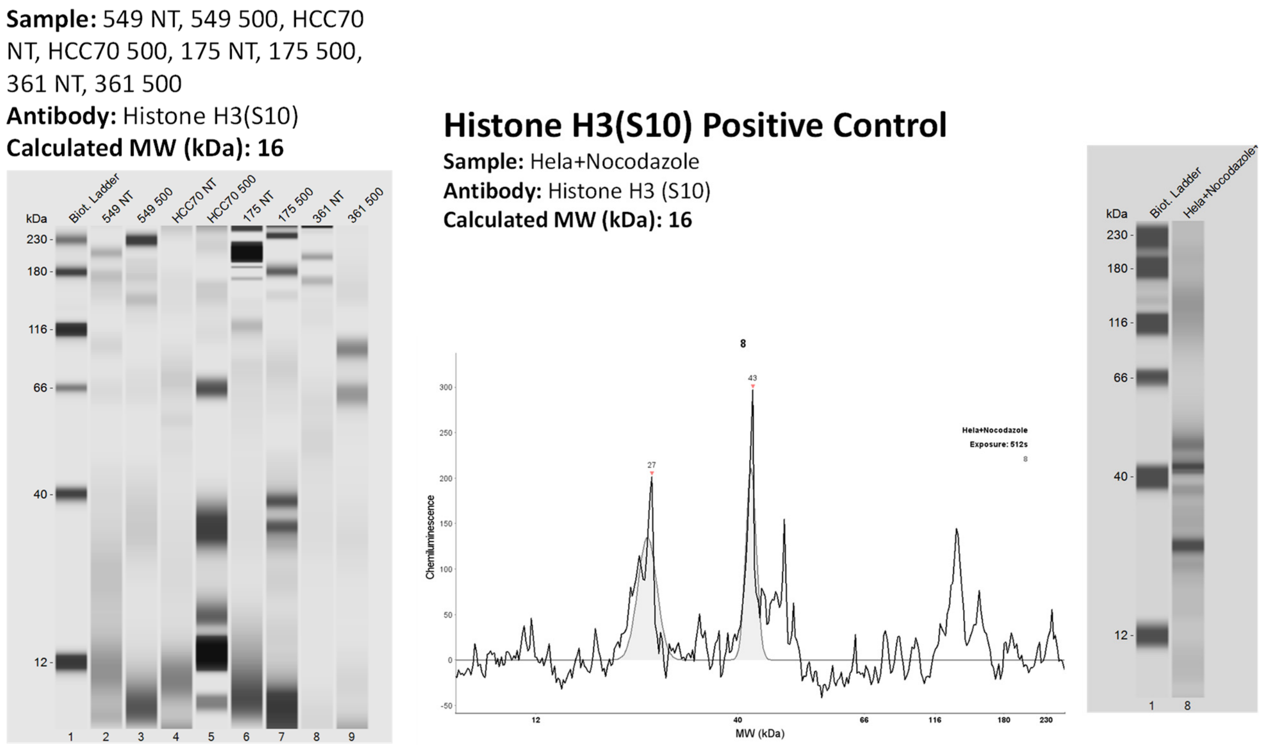

Western immunoblot of β-actin to determine the protein load in lysates from BCa cells untreated (NT) and IBCar-treated (500 nM IBCar, 24 h) lysates.

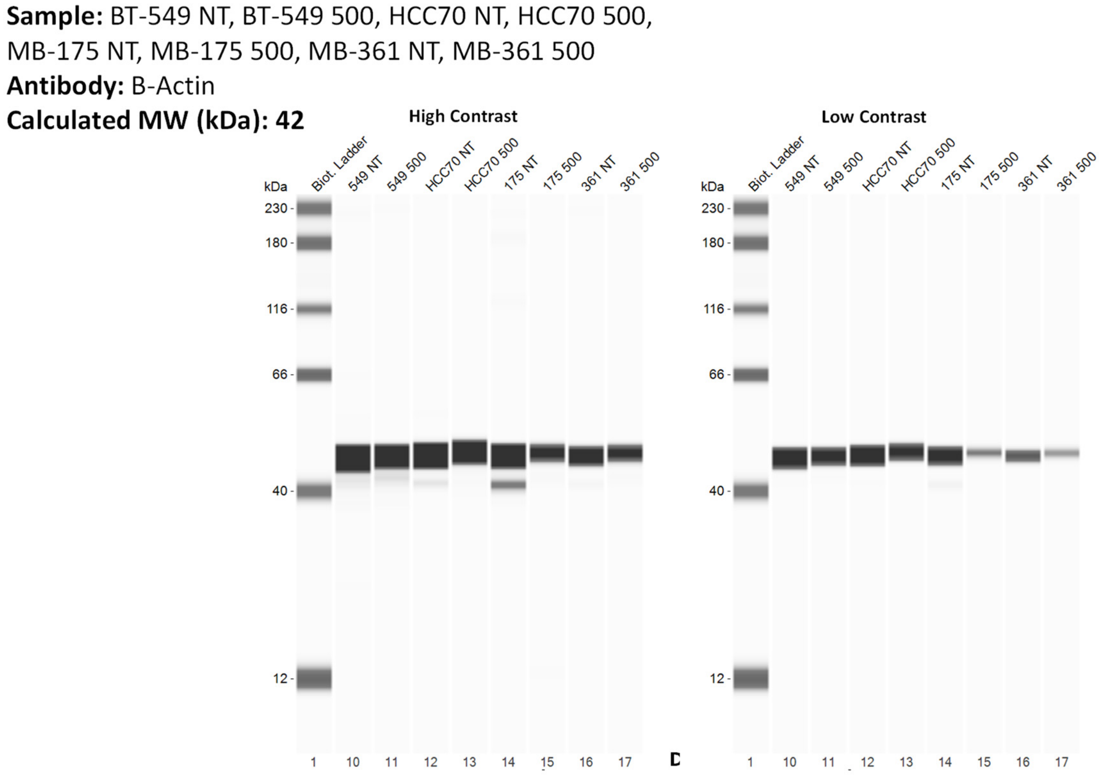

Figure S6. **Confirmatory Western immunoblot of Cas-8 in lysates** from BCa cells untreated (NT) and IBCar-treated (500 nM IBCar, 48 h) using rabbit anti-human, mouse, rat-Cas-8 antibodies PA5-87373. Total protein load ~120 µg/well was used to increase the probability to detect low levels of Cas-8 and to determine in Cas-8 is present in untreated BCa cells BT549, HCC70 and MB175.

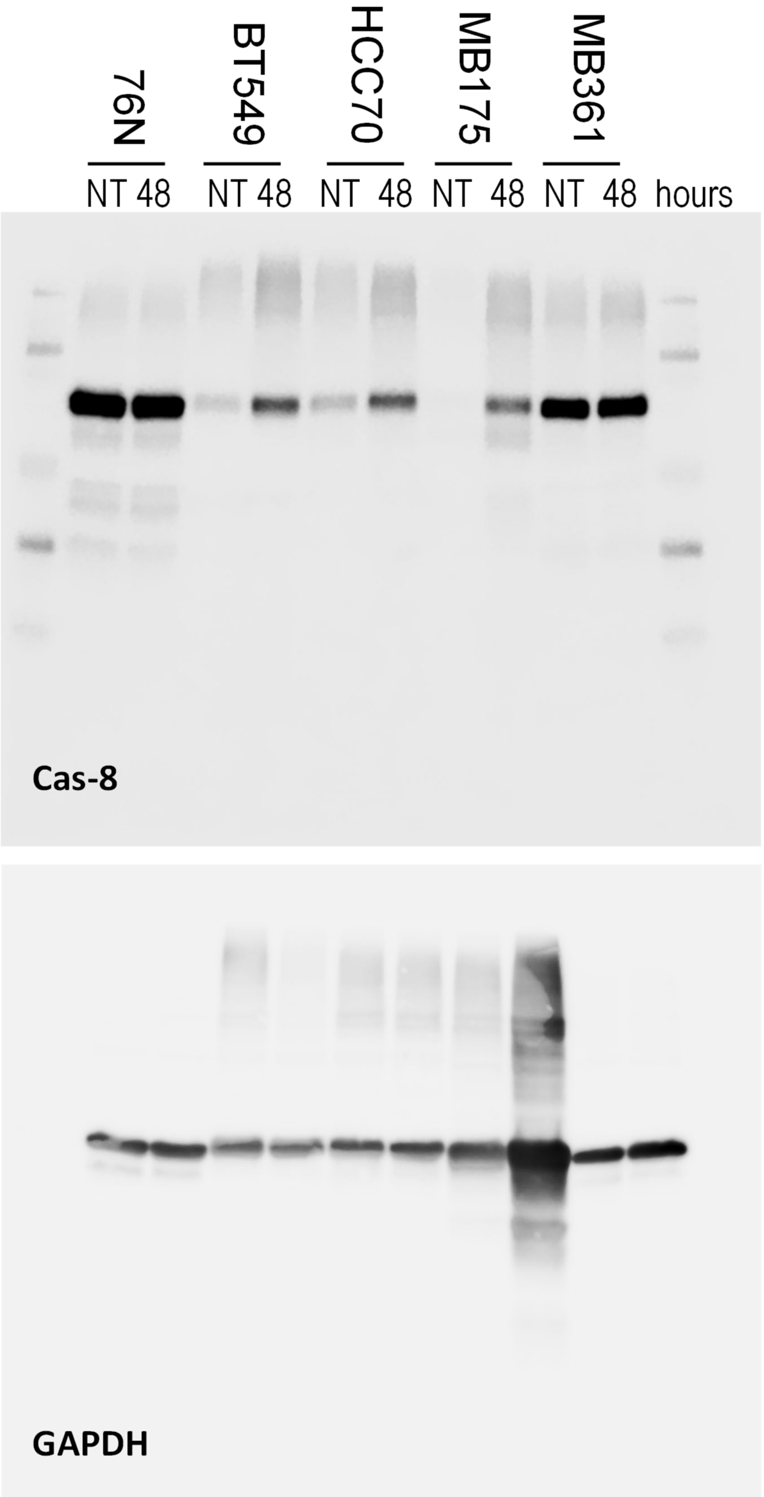

Figure S7. **Human HSP arrays** (shown in Figure 13) of lysates from BCa cells untreated (NT) and IBCar-treated (500 nM IBCar, 24 h).

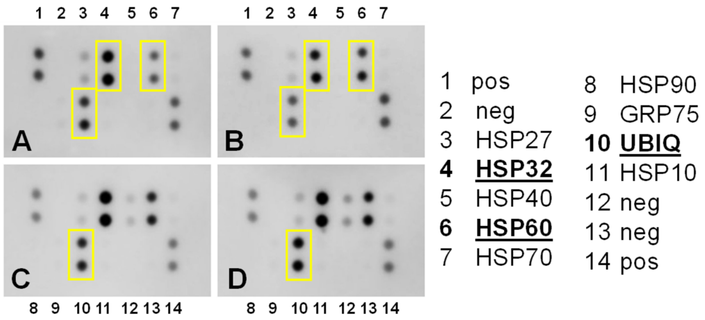

A. Untreated 76N cells. **B**. IBCar treated 76N cells. **C**. Untreated MB468 cells. D. IBCar-treated MB468 cells. UBIQ = RPS27a.

Table S5. Analyses of MDA-MB-468 xenograft growth curves and Kaplan-Meier survival curves.

MDA-MB-468
NT vs. IBCar
Unpaired t test with Welch's correction
P value<0.0001
Significantly different (P<0.05)? Yes
Two-tailed P value
Welch-corrected t, df t=6.604, df=12.93
F test to compare variances
F, DFn, Dfd 11.34, 11, 11
P value = 0.0003
Significantly different (P < 0.05)? Yes

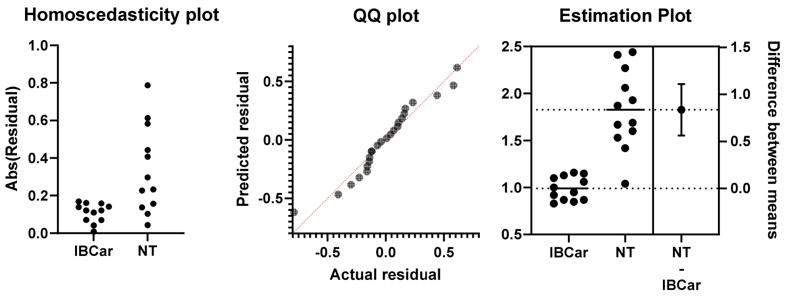

Comparison of Survival Curves
Gehan-Breslow-Wilcoxon test
Chi square 9.276
df 1
P value 0.0023
Are the survival curves sig different? Yes

Hazard Ratio (logrank) A/B B/A
Ratio (and its reciprocal) 0.1368 7.309
95% CI of ratio 0.04052 to 0.4620 2.165 to 24.68

Log-rank (Mantel-Cox) test
Chi square 10.15
df 1
P value 0.0014
Are the survival curves sig different? Yes

Hazard Ratio (Mantel-Haenszel) A/B B/A
Ratio (and its reciprocal) 0.1211 8.257
95% CI of ratio 0.03305 to 0.4438 2.253 to 30.26

Figure S8. Longitudinal changes in tumor volume in control mice and IBCar-treated mice.

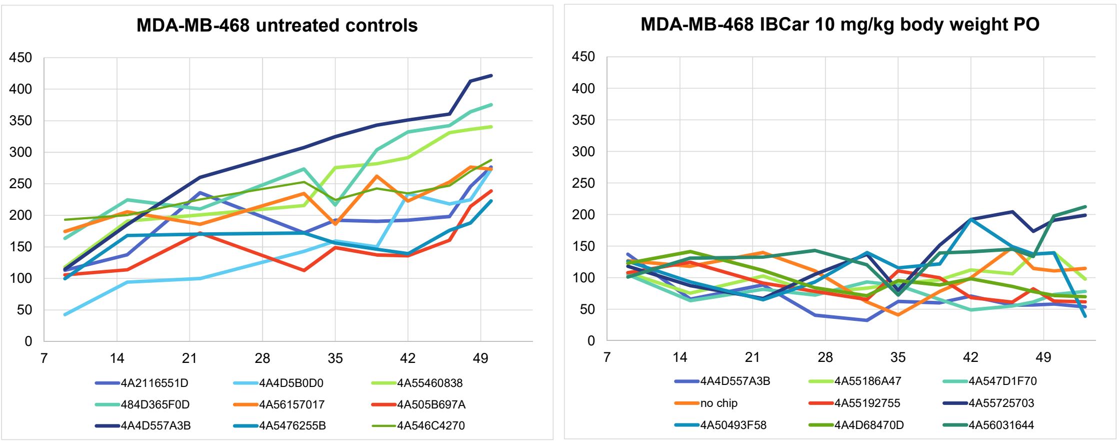

Figure S9. **Nuclear fragmentation in normal and BCa cells treated with IBCar.** Blue arrowheads indicate fragmented nuclei and micronucleation. Only MB175 cells have a significant number of fragmented nuclei.

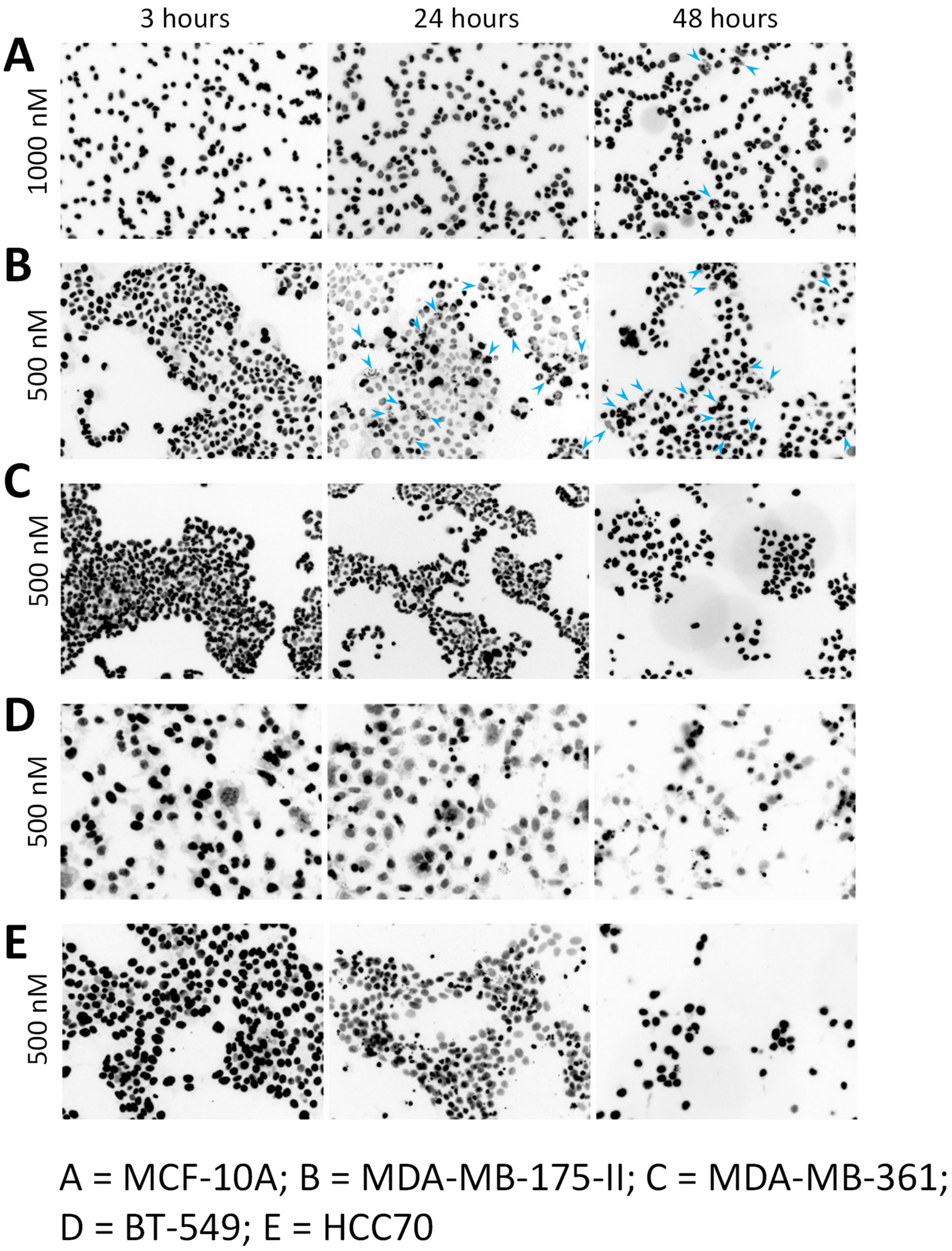

Figure S10. **Determination of the basal levels of activated Cas-3/7 in normal breast and BCa cells.**

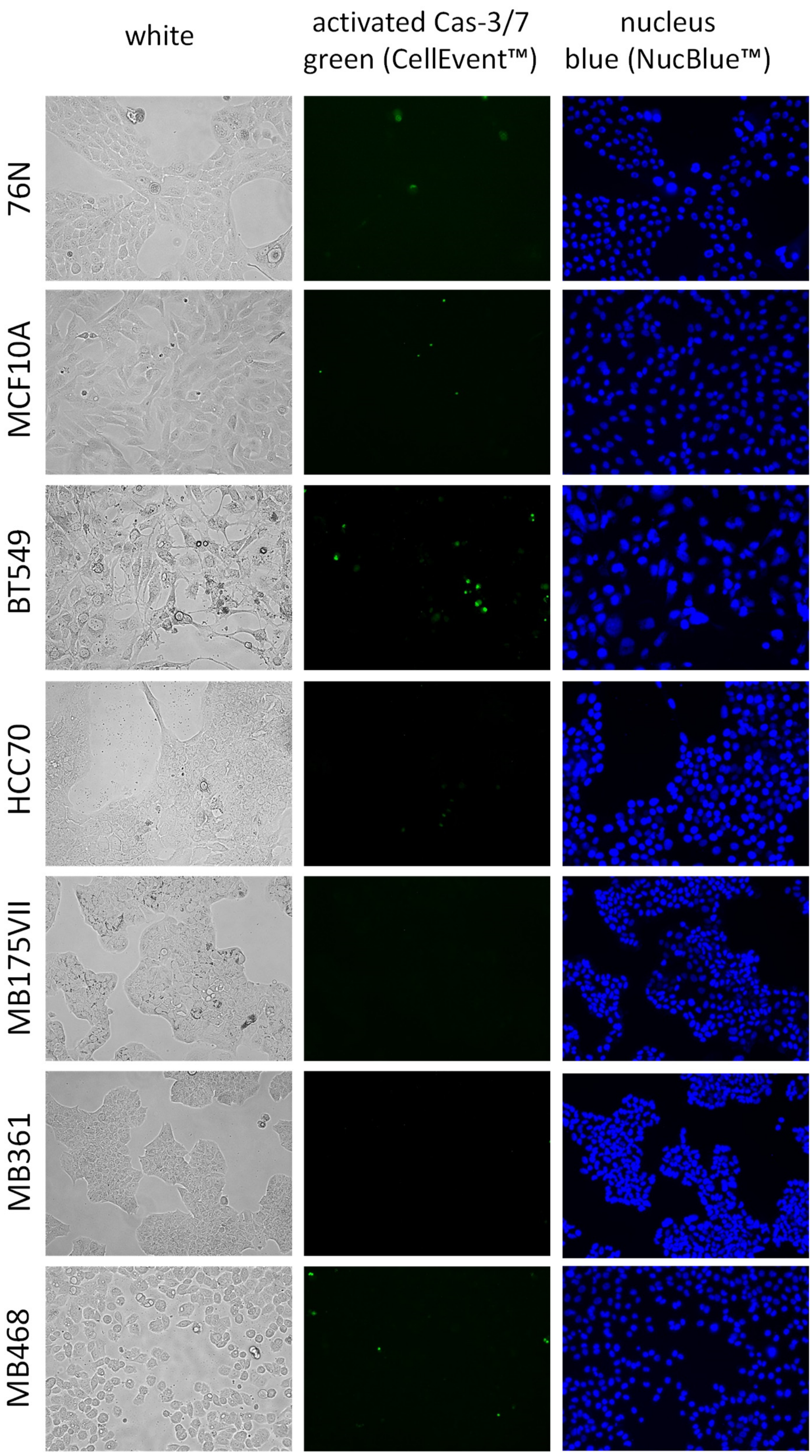

Figure S11. **Activated Cas-3/7 populations in HCC70 and MB468 cells treated with 100 nM and 500 nM IBCar**.

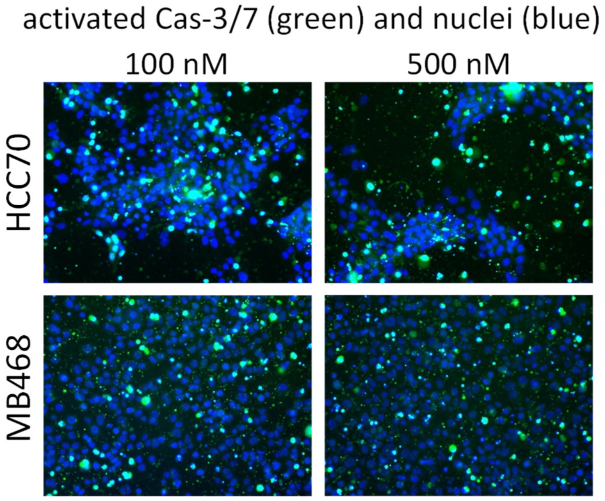

Table S6. Hematology of J:NU mice bearing human breast cancer xenografts.

parameter	units	MDA-MB-468 IBCar	MDA-MB-468 control	Jax data J:NU female
White blood cell count (wbc)	10^3 cells/μL	4.9	4.89	6.3
Lymphocyte count (Lym)	10^3 cells/μL	3.62	4.17	4.5
Monocyte count (Mon)	10^3 cells/μL	0.21	0.33	0.19
Neutrophil count (Neu)	10^3 cells/μL	1.07	0.39	1.4
Percent lymphocytes (Lym%)	%	73.9	85.3	71.4
Percent monocytes (Mon%)	%	4.3	6.8	3.0
Percent neutrophils (Neu%)	%	21.8	7.9	16.0
Red blood cell count (Rbc)	10^6 cells/μL	10.92	10.85	9.9
Hemoglobin (Hb)	g/dL	15.3	15.5	15.6
Hematocrit (Hct)	%	48.92	51.2	47.0
Mean corpuscular volume (MCV)	fL	45.0	47.0	47.5
Mean corpuscular Hb (MCH)	pg	14.0	14.3	15.8
MCH concentration (MCHC)	g/dL	31.3	30.3	33.3
Rbc distribution width (RDW-cv)	%	20.5	19.4	na
Rbc distribution width (RDW-sd)	fL	32.8	33.8	na
Platelet count (Plt)	10^3 cells/μL	696.0	504.0	1385
Mean Plt volume (MPV)	fL	6.7	6.5	6.4
Plt hematocrit (Pct)	%	0.47	0.33	na
Plt distribution width (PDW-cv)	5	31.9	30.5	na
Plt distribution width (PDW-sd)	fL	8.8	8.0	na

Table S7. Serum chemistry of female J:NU mice bearing human breast cancer xenografts.

		MDA-MB-468	
		**IBCar**	**control**
**parameter**	units	average (std dev)	average (std dev)
Albumin (ALB)	g/dL	4.7 (0.05)	5.3 (0.4)
Alkaline phosphatase (ALP)	U/L	90.0 (11.1)	95.5 (10.6)
Alanine aminotransferase (ALT)	U/L	27.0 (2.9)	38.0 (14.1)
Amylase (AMY)	U/L	730 (21)	1040 (275)
Bilirubin, total (total bil)	mg/dL	0.2 (0.1)	0.3 (0.0)
Bood urea nitrogen (BUN)	mg/dL	17.8 (2.2)	14.0 (0.0)
Calcium (Ca)	mg/dL	11.3 (0.9)	12.4 (0.1)
Phosphorus (PHOS)	mg/dL	8.1 (0.3)	10.0 (0.1)
Creatinine (CRE)	mg/dL	0.3 (0.1)	0.5 (0.1)
Glucose (GLU)	mg/dL	199.3 (17.9)	247 (30)
Sodium (Na^+)	mmol/L	168.8 (2.5)	168 (2.8)
Potassium (K^+)	mmol/L	8.5 (0.0)	8.5 (0.0)
Total protein (TP)	g/dL	5.5 (0.2)	6.4 (0.6)
Globulin (GLOB)	g/dL	0.8 (0.2)	1.2 (0.1)

Figure S12. Microtubules in untreated HCC70 breast cancer cells.

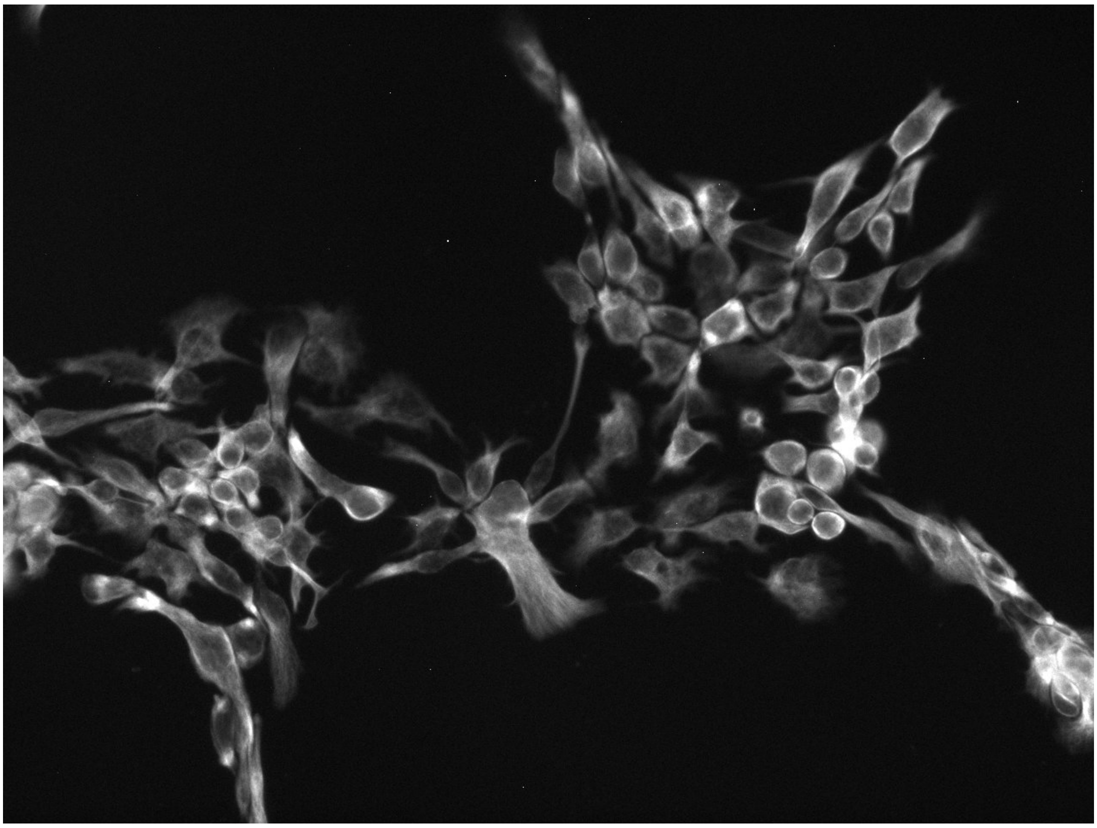

Figure S13. Remnants of microtubules in HCC70 breast cancer cells treated with IBCar.

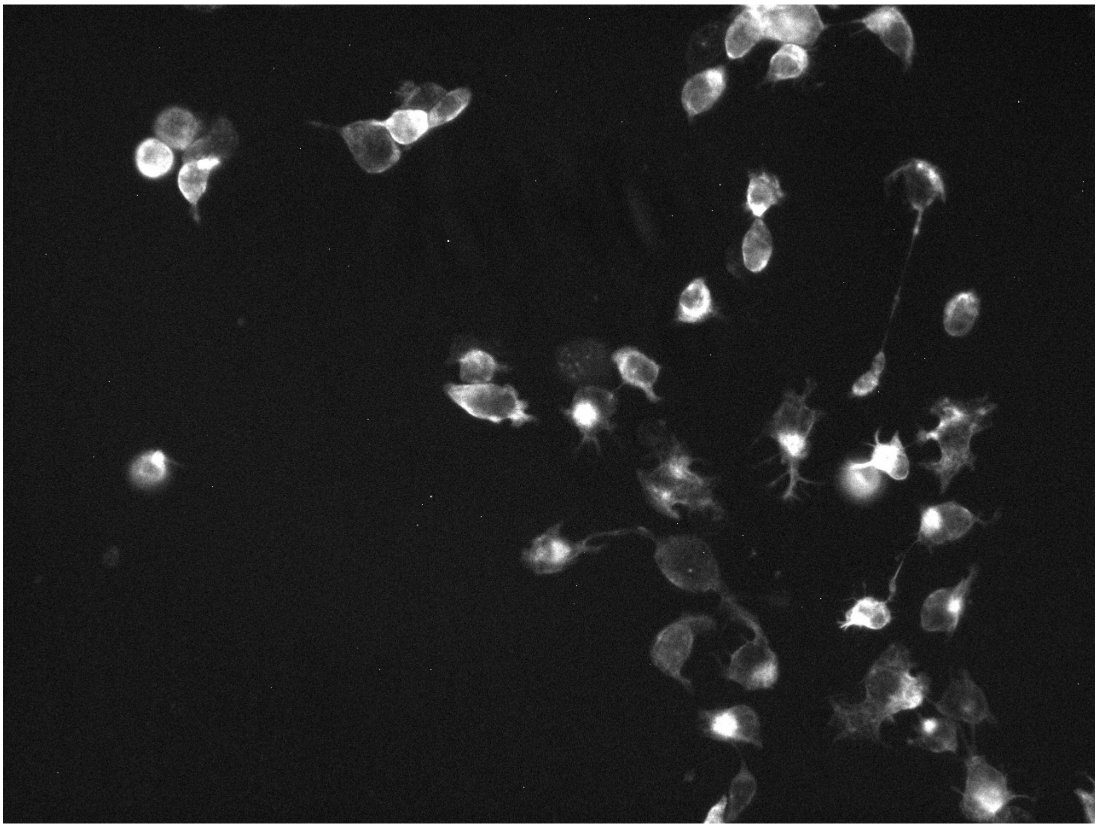

Figure S14. Microtubules in untreated 76N normal breast cells.

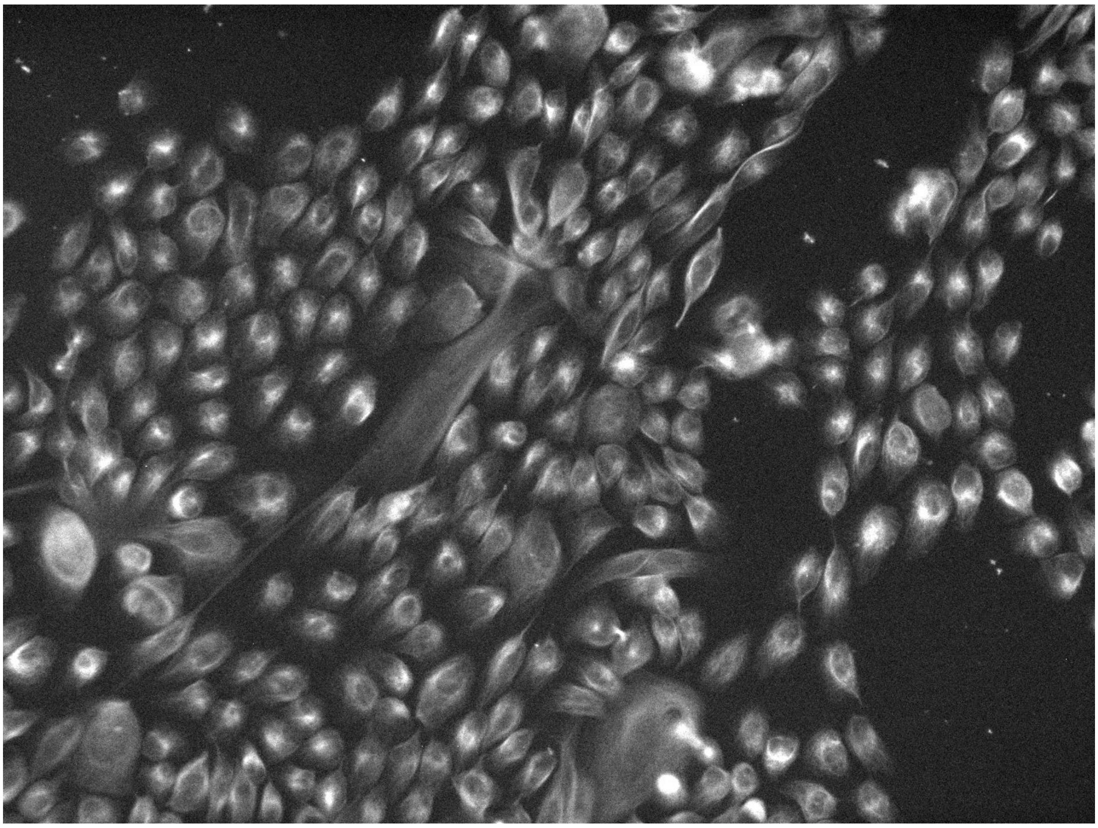

Figure S15. Microtubules in 76N normal breast cell treated with IBCar.

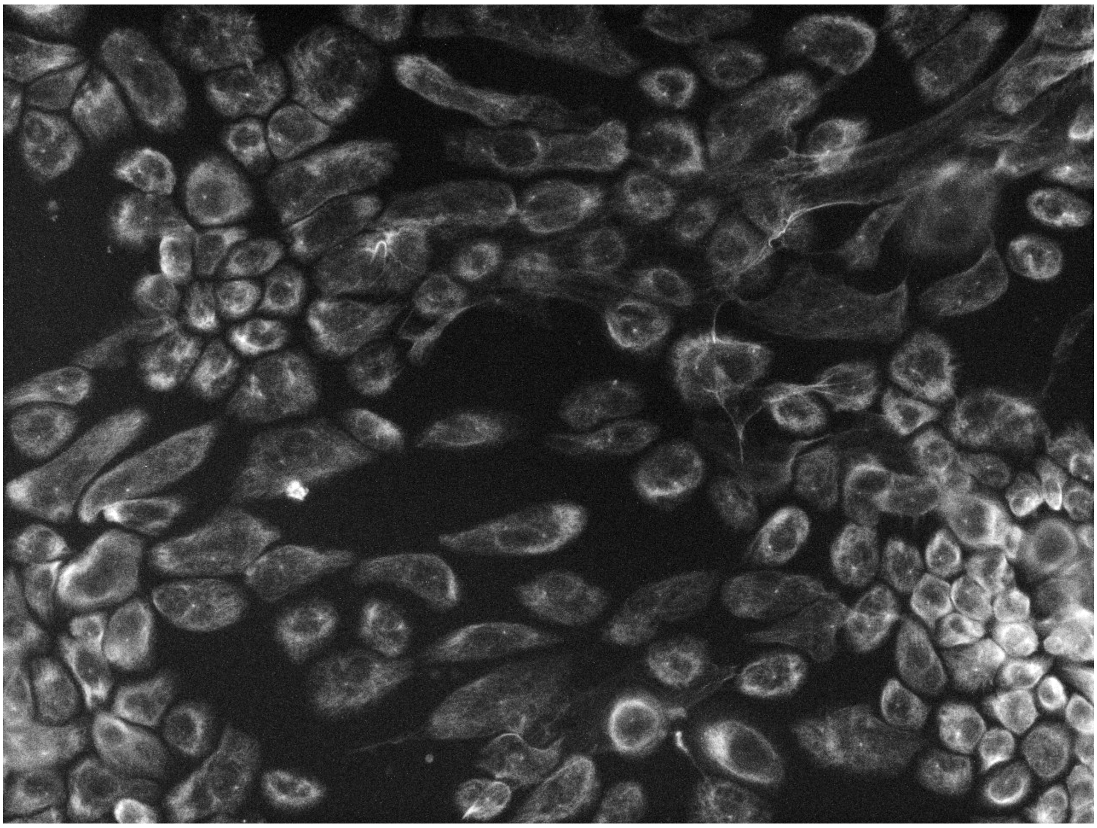

Table S8. GI_{50} concentrations of mebendazole, vincristine, and IBCar determined by the NCI DTP in several breast cancer cell lines.

cell line	Mebendazole [nM]	Vinolerbine [nM]	IBCar [nM]
BT-549	3,273.4	43.0	74.9
HS 578T	199.5	27.7	38.9
MCF7	204.2	nd	35.9
MDA-MB-231	1,253.1	21.1	101.0
MDA-MB-468	121.3	nd	27.9

Table S9. Tumor growth curve analyses in MMTV-PyMT mice.

NT vs. IBCar
Unpaired t test
P value <0.0001
Significantly different (P < 0.05)? Yes
One- or two-tailed P value? Two-tailed

How big is the difference?
Difference between means (B - A) ± SEM 1331 ± 201.9
95% confidence interval 920.0 to 1743
R squared (eta squared) 0.5760

Table S10. Statistical comparison of tumor weights extirpated from MMTV-PyMT mice at the age of 81 days.

NT vs. IBCar
Unpaired t test
P value 0.009
Significantly different (P < 0.05)? Yes
One- or two-tailed P value? Two-tailed

How big is the difference?
Difference between means (B - A) ± SEM 1.685 ± 0.4058
95% confidence interval 0.6420 to 2.729
R squared (eta squared) 0.7752

Table S11. Statistical comparison of survival curves in MMTV-PyMT transgenic mice.

Gehan-Breslow-Wilcoxon test
Chi square 8.649
P value 0.0033
Are the survival curves sig different? Yes

Log-rank (Mantel-Cox) test
Chi square 9.090
P value 0.0026
Are the survival curves sig different? Yes

Median survival
IBCar Undefined
control 74.00

Hazard Ratio (Mantel-Haenszel) A/B B/A
Ratio (and its reciprocal) 0.05359 18.66
95% CI of ratio 0.007995 to 0.3592 2.784 to 125.1

Hazard Ratio (logrank) A/B B/A
Ratio (and its reciprocal) 0.1484 6.740
95% CI of ratio 0.02800 to 0.7862 1.272 to 35.72
