# Supplementary material for: IBCar: Potent Orally Bioavailable Methyl N-[5-(3′-Iodobenzoyl)-1H-Benzimidazol-2-yl]Carbamate for Breast Cancer Therapy
Source: Cancers (Basel). 2025 Jul 30;17(15):2526. doi: 10.3390/cancers17152526 (PMC12346495; doi:10.3390/cancers17152526)

p-cdc2 (Figure 3D)

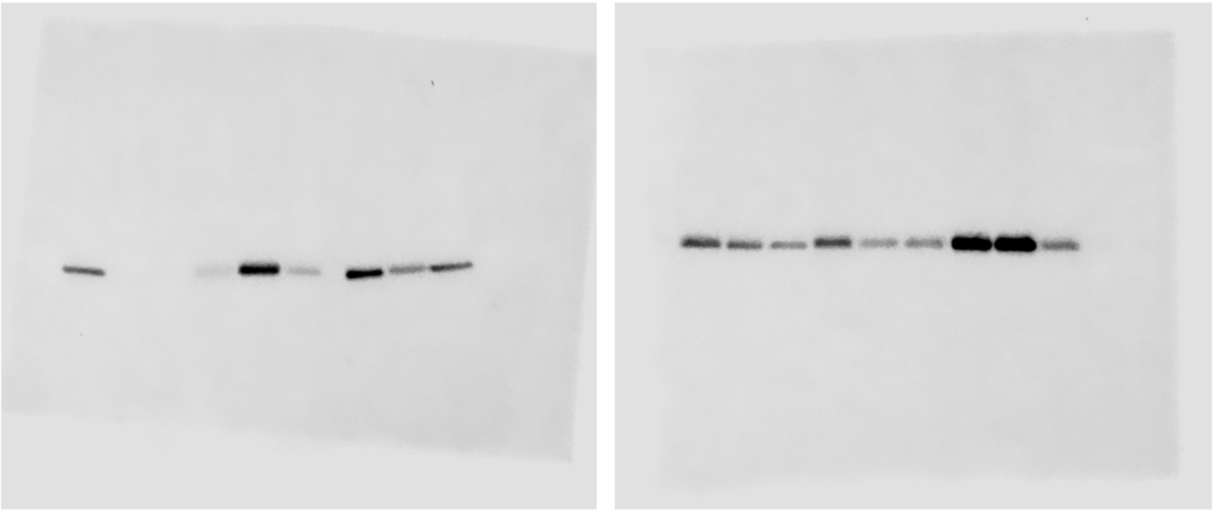

Cas-3 (Figure 4C)

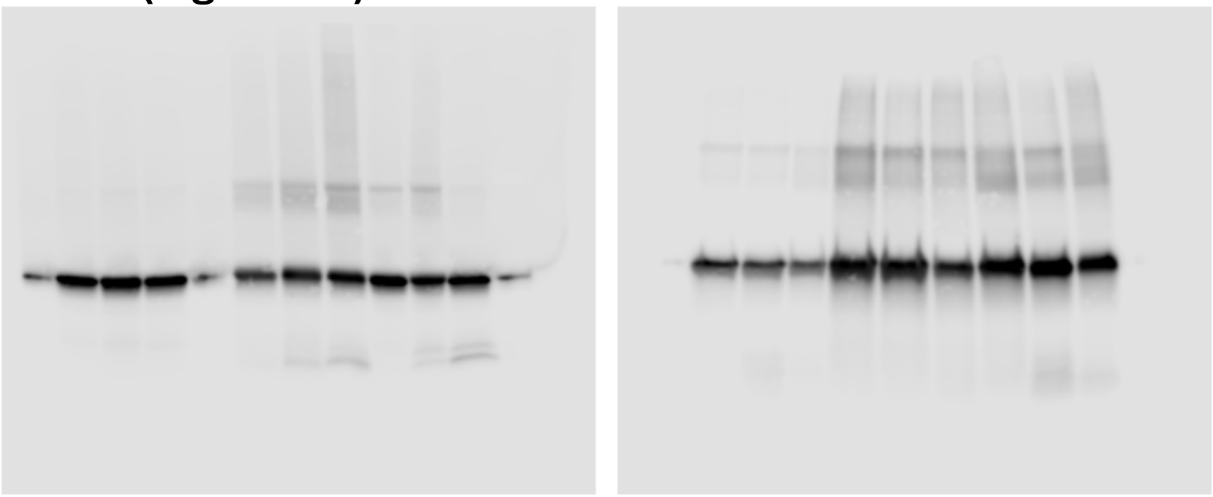

cleaved Cas-3 (Figure 4C)

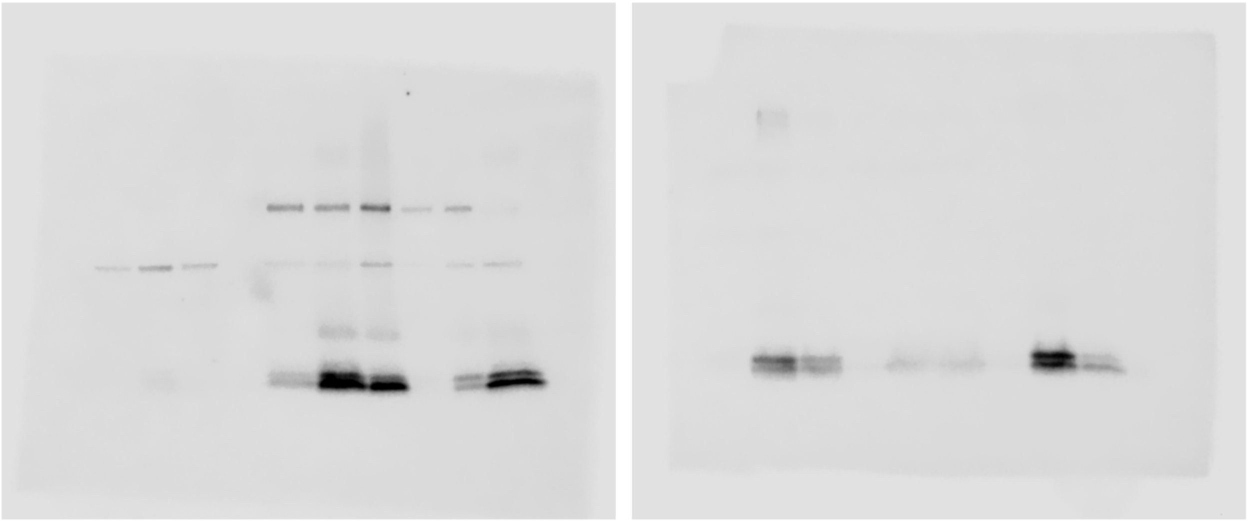

Cas-8 (Figure 5A)

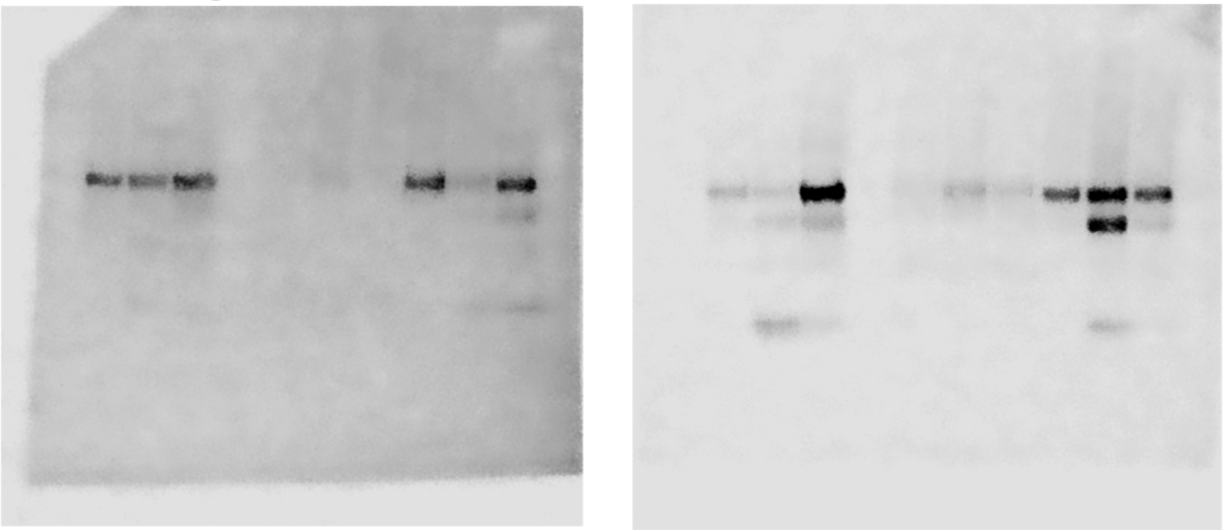

MLKL (Figure 5B)

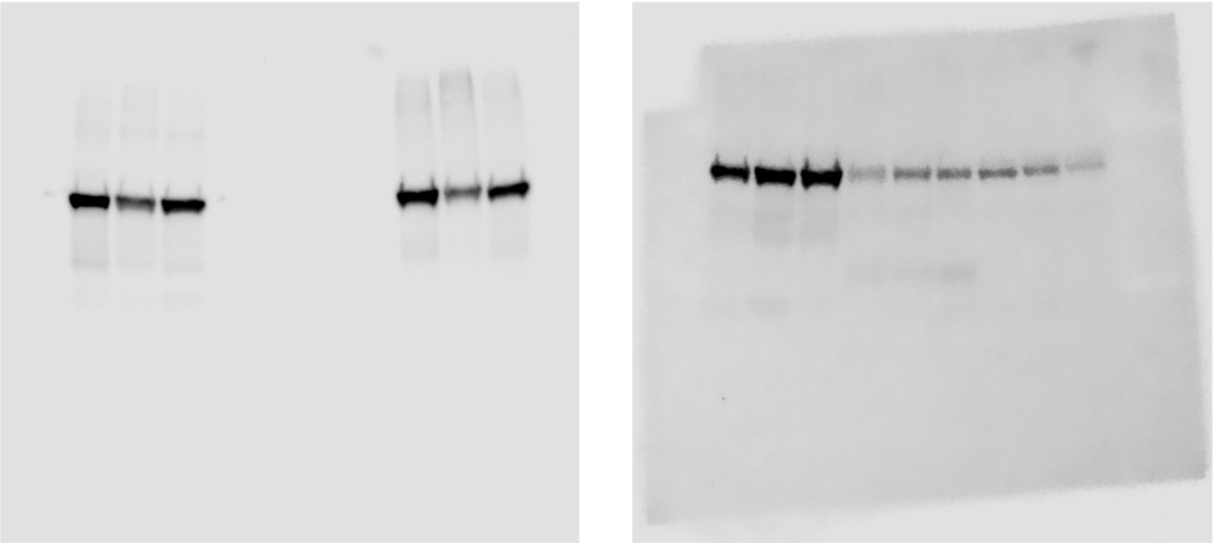

RIP3 (Figure 5C)

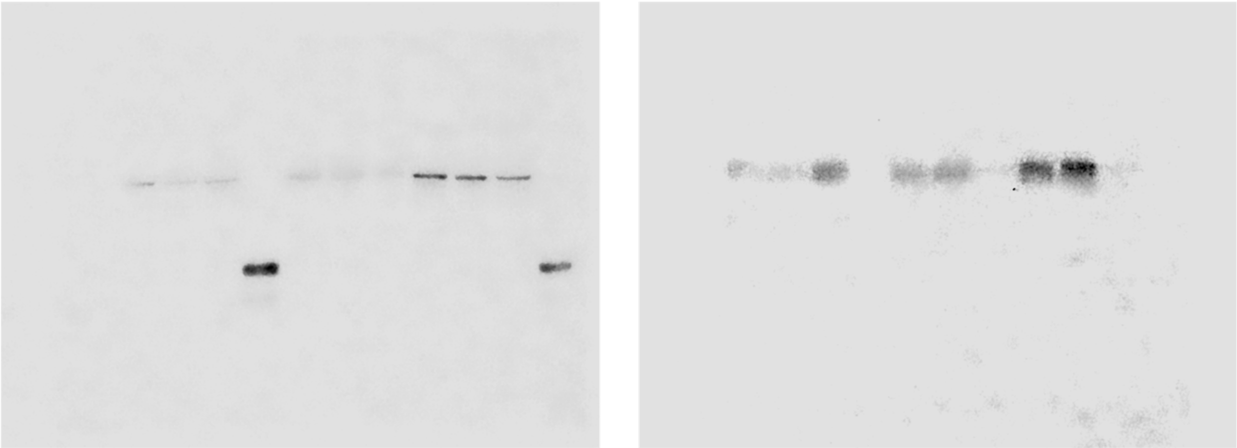

cleaved Cas-8 (Figure 6)

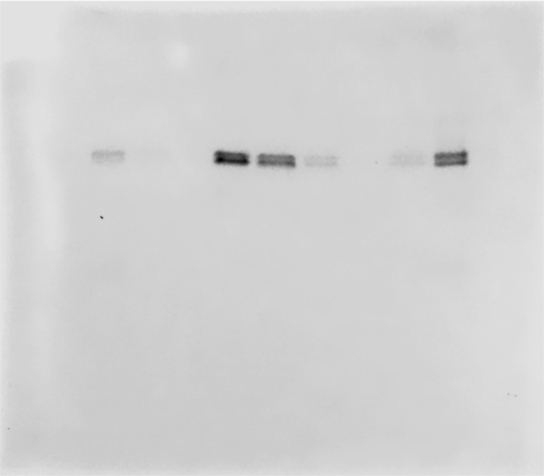

RIP1 (Figure 7)

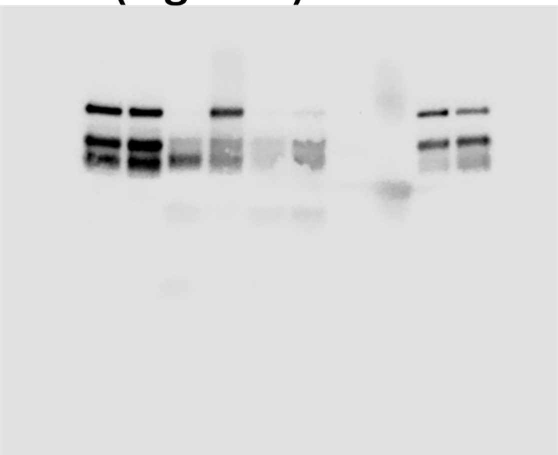

p-RIP1 (Figure 7)

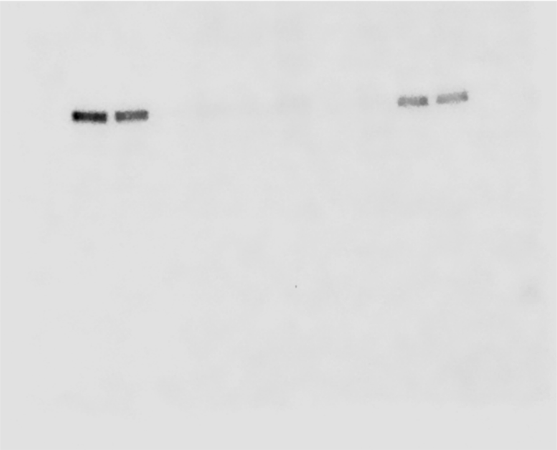

BiP (Figure 11A)

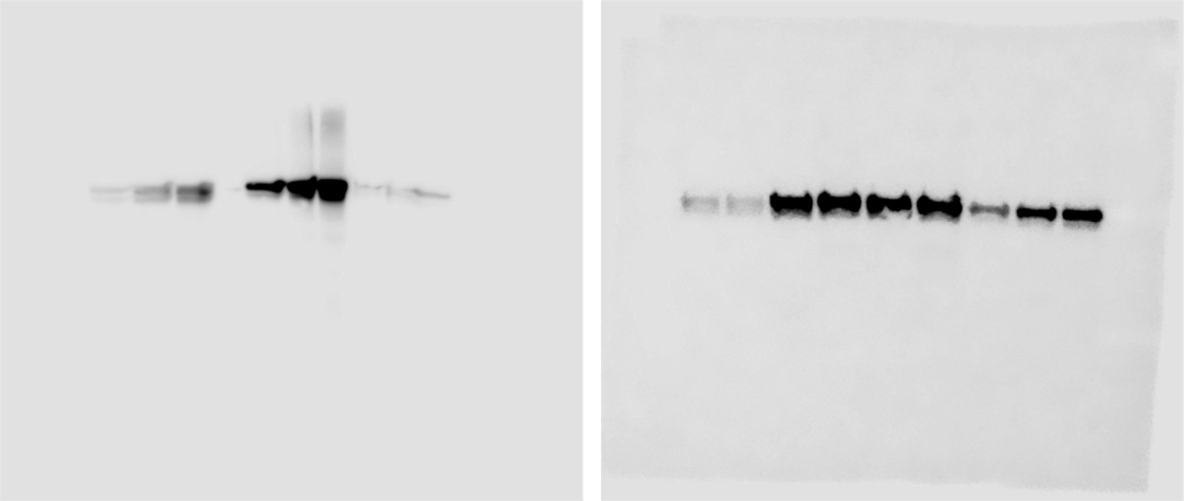

Calnexin (Figure 11A)

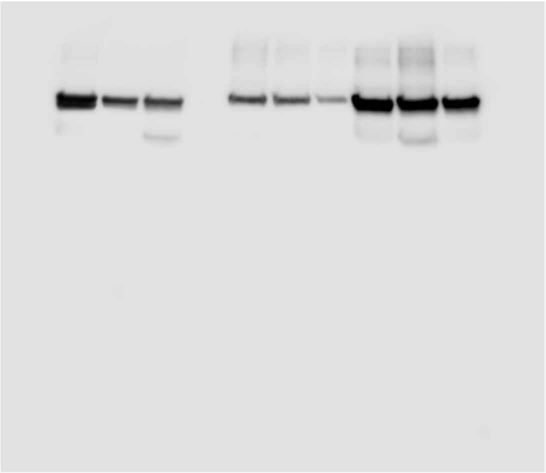

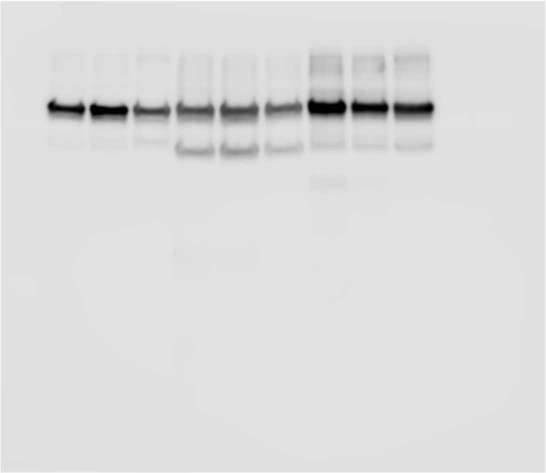

PERK (Figure 12A)

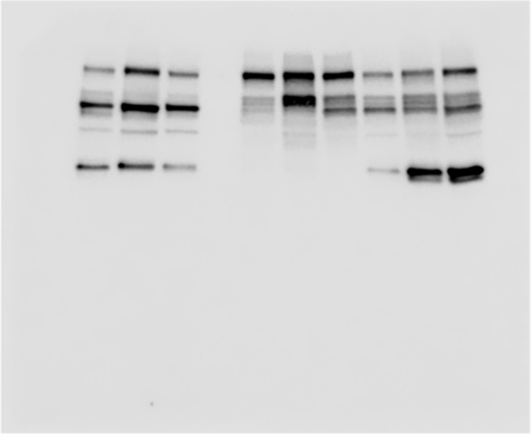

eIF2α (Figure 12C)

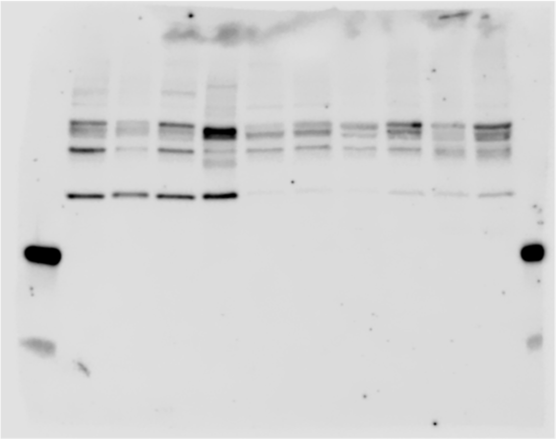

IRE1α (Figure 12C)

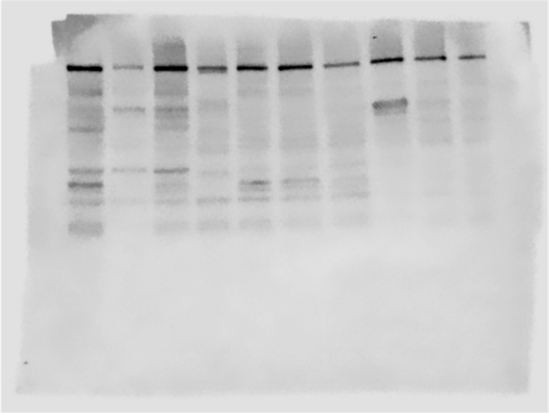

p-IRE1α (Figure 12C)

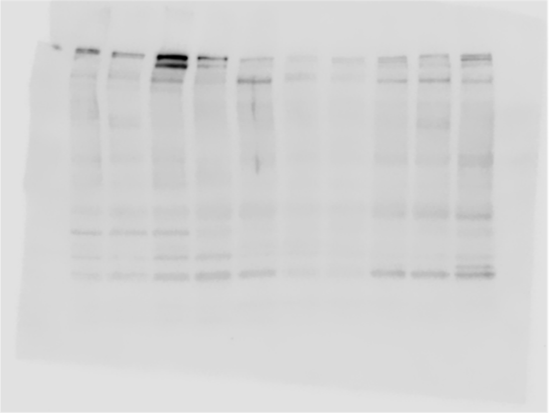

ER-α (Figure 15G)

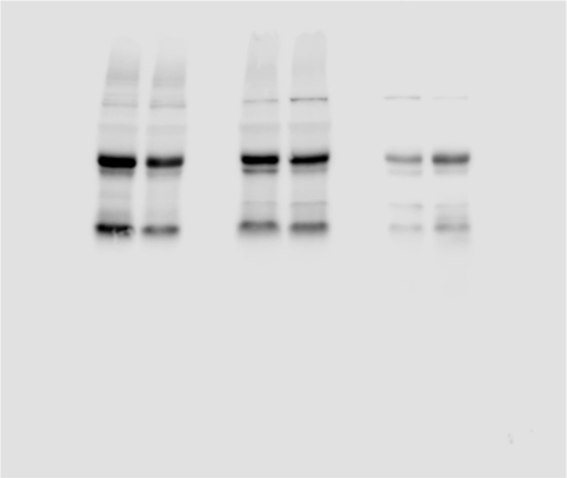

PR (Figure 15G)

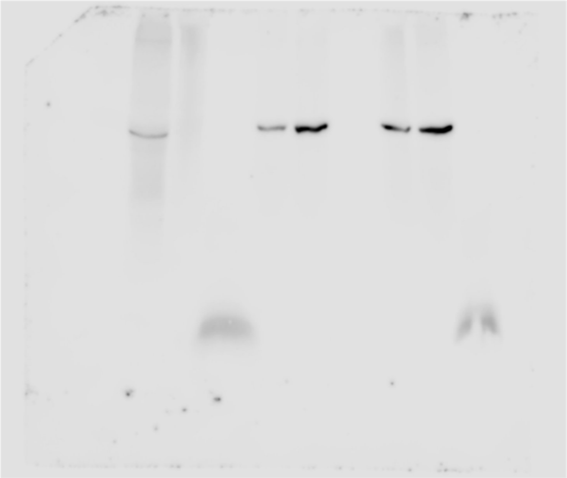

Figure S4

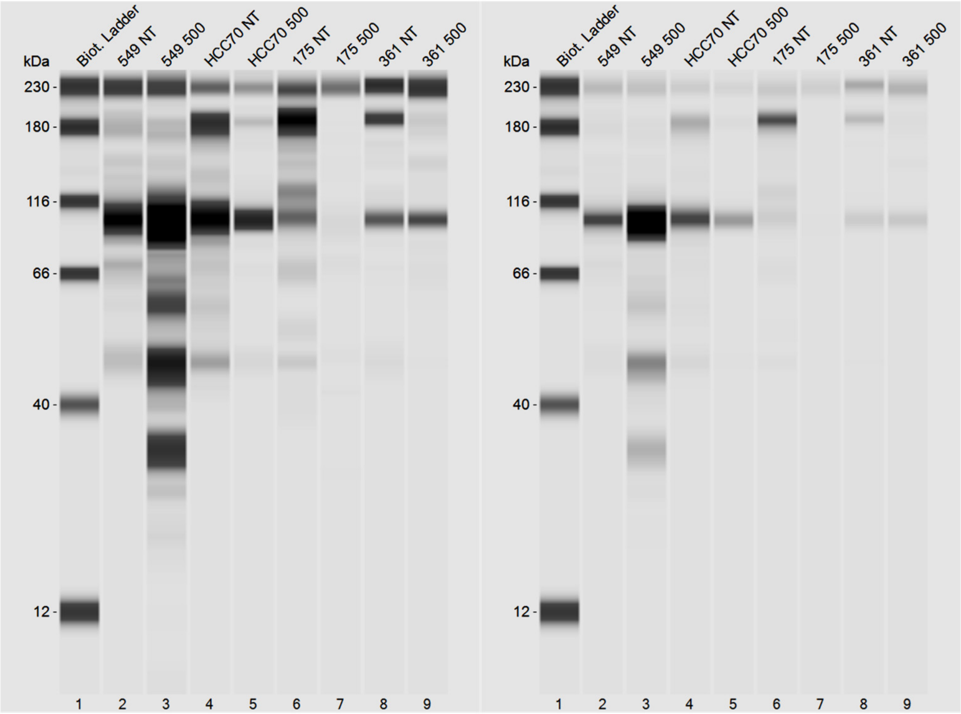

Figure S5

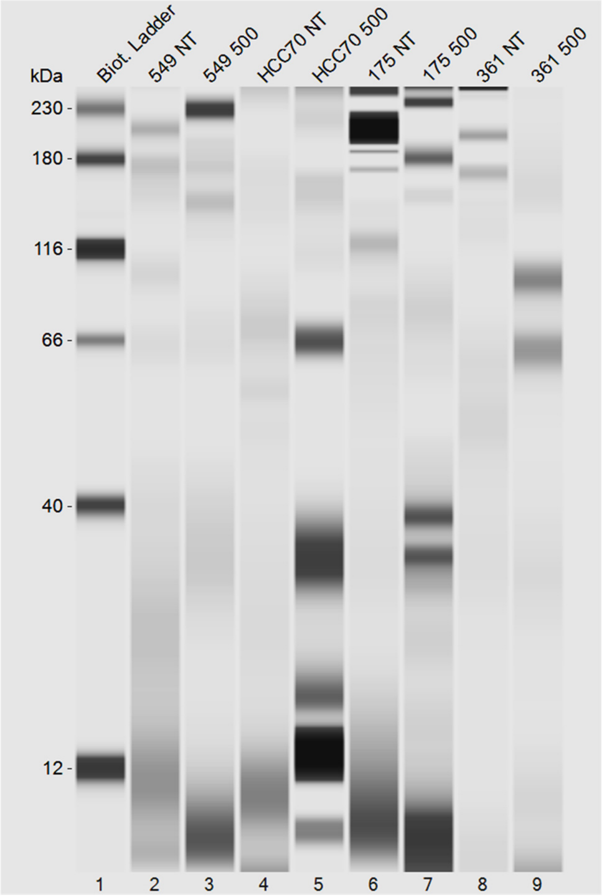

Figure S6

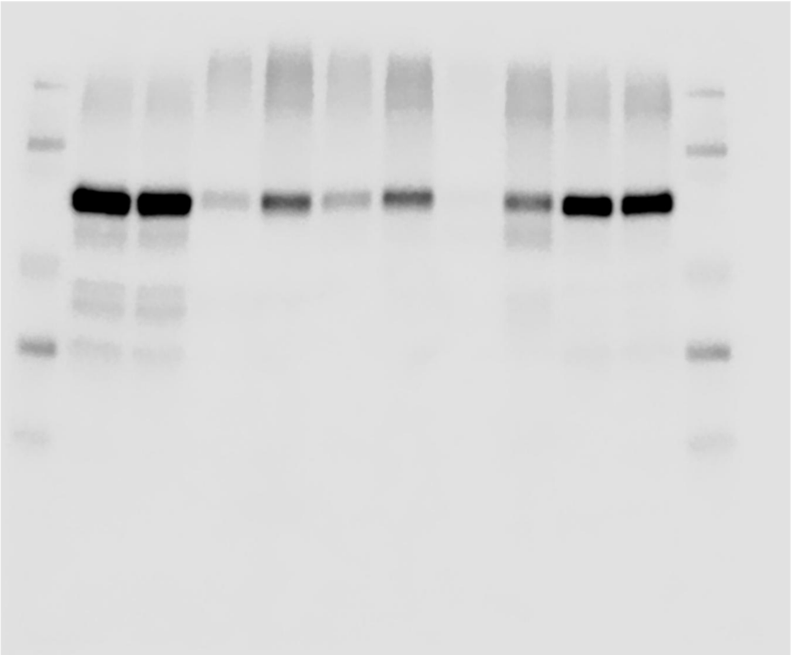

Supplement: Supplementary file 1 [file cancers-17-02526-s001.zip › uncropped Western blots.pdf]
